# Approaches for the treatment of perforated peptic ulcers: a network meta-analysis of randomized controlled trials

**DOI:** 10.1007/s00423-025-03848-9

**Published:** 2025-09-05

**Authors:** Elisabeth Wadewitz, Juliane Friedrichs, Maurizio Grilli, Johannes A. Vey, Samuel Zimmermann, Yoshiaki Sunami, Jörg Kleeff, Ulrich Ronellenfitsch, Johannes Klose, Artur Rebelo

**Affiliations:** 1 Department of Visceral, Vascular and Endocrine Surgery, University Hospital Halle (Saale), Martin Luther University Halle Wittenberg, Halle, Germany; 2 Library of the Medical Faculty Mannheim, Mannheim, Germany; 3 Institute of Medical Biometry, University Hospital Heidelberg, Heidelberg, Germany; 4 Department of Visceral, Vascular and Endocrine Surgery, University Hospital Halle (Saale), Martin-Luther- University Halle-Wittenberg, Ernst-Grube-Str. 40, 06120 Halle (Saale), Germany

**Keywords:** Perforated peptic ulcers, Treatment approaches, Network meta-analysis, Emergency surgery

## Abstract

**Purpose:**

This network meta-analysis (NMA) aims to evaluate surgical and alternative treatment strategies for perforated peptic ulcers (PPU) with respect to mortality and other clinically relevant outcomes.

**Methods:**

An NMA was conducted in accordance with PRISMA guidelines to assess treatment approaches for PPU. Randomized controlled trials (RCT) were identified through systematic searches of PubMed/MEDLINE, Cochrane Library, Embase, CINAHL, ClinicalTrials.gov, and ICTRP databases. Outcomes were analyzed using standardized mean differences (SMDs) for continuous data and odds ratios (ORs) for binary data, both presented with 95% confidence intervals (CI) in a network meta-analysis framework.

**Results:**

Sixteen studies comprising 1,259 patients were included in this NMA. The laparoscopic approach demonstrated significantly reduced mortality (OR 0.36, 95% CI 0.17–0.75, *p* = 0.0065) and postoperative complications, including wound infections (OR 0.15, 95% CI 0.08–0.27, *p* < 0.0001) and ileus (OR 0.33, 95% CI 0.18–0.59), compared to the open surgical approach.

**Conclusions:**

This NMA, particularly the pairwise analysis, confirms the significant advantages of laparoscopic over open surgery, reinforcing its status as the gold standard for PPU. The potential benefits of alternative approaches, are inconclusive due to insufficient evidence.

**Supplementary Information:**

The online version contains supplementary material available at 10.1007/s00423-025-03848-9.

## Introduction

Peptic ulcer disease affects millions of patients worldwide, with a global lifetime prevalence estimated at 5–10% [1]. Perforated peptic ulcer (PPU) is a severe complication of peptic ulcer disease associated with high morbidity and mortality, often necessitating emergency surgery [2].

In 1946, Taylor described a series of 28 conservatively treated PPU cases, reporting a mortality rate of 14% [3]. Advances in minimally invasive techniques have introduced combined approaches for managing PPU. In the laparoscopy-endoscopy approach, perforations are endoscopically closed with a stent, while lavage and drainage are performed laparoscopically [4]. Similarly, the radiologic-endoscopic method employs endoscopic stent placement with lavage and fluid drainage via a radiological placed drain [5].

Currently, the most common surgical techniques are open and laparoscopic surgery. The debate over their relative merits has been extensive. Laparoscopy has shown potential advantages, including reduced mortality, morbidity, and shorter hospital stay [6–10]. However, some studies report no significant differences between laparoscopic and open surgery regarding overall postoperative complications and mortality, concluding that both methods are comparable for PPU repair [11]. In emergency settings, laparoscopy may present disadvantages, such as longer operative times and the requirement of specific surgical expertise [10, 12].

PPU remains a critical health issue due to its significant mortality, morbidity, and financial burden [13]. This meta-analysis aims to compare and evaluate different treatment approaches for PPU concerning mortality and other clinically relevant outcomes.

## Methods and analysis

Literature search and data analysis were conducted in accordance with the PRISMA (Preferred Reporting Items for Systematic Reviews and Meta-Analyses) guidelines [1]. This study was registered in the PROSPERO database (ID: CRD42023482932) [2] and its protocol was published a priori [3].

### Search strategy

**Fig. 1:**
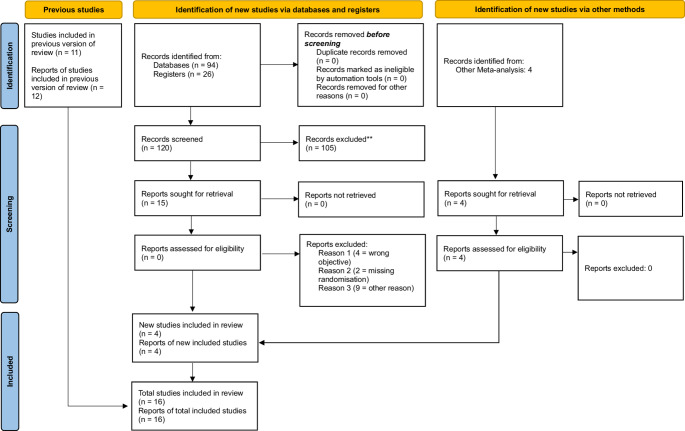
PRISMA 2020 flow chart

We searched the databases Pubmed/MEDLINE, Cochrane Library, Embase, CINAHL, ClinicalTrials.gov and ICTRP. The search strategy for each database can be found in the online supplemental material 1. The literature search included all studies that were published between inception of the respective databases and December 2023. We repeated the search in November 2024. Two authors (JF and EW for the initial search, EW and AR for the updated search) independently assessed titles and abstracts of eligible studies. These studies were coded as “include”, “maybe” or “exclude”. For studies marked as “include” or “maybe”, full texts were retrieved and assessed for inclusion. In cases of discrepancies between the reviewers, a third reviewer (AR) was involved in order to reach a consensus. For the updated research, JF served as the third author in case no consensus could be reached. The study selection process is shown in Fig. 1, according to the PRISMA 2020 statement.

## Inclusion and exclusion criteria

**Fig. 2:**
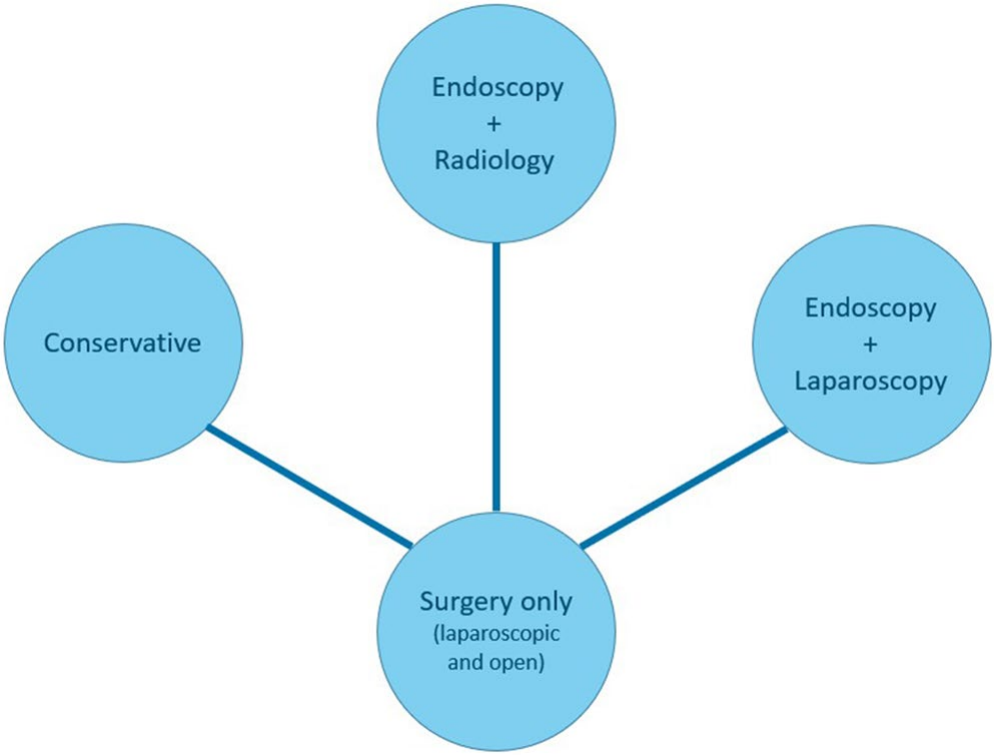
Network graph of direct evidence between interventions

For inclusion we considered RCTs which compared at least two treatment approaches for PPU. The following interventions were included: open surgical treatment (A), laparoscopic treatment (B), combined endoscopic and laparoscopic treatment (C), combined endoscopic and interventional radiologic treatment (D), conservative treatment (E) (Fig. 2). We included studies on patients with confirmed diagnosis of PPU regardless of age, symptoms or medical history. Non-randomized studies, letters, comments, case reports or case series were excluded from consideration. There were no language restrictions.

### Data collection

Two authors (JF, EW) independently extracted the data from the included studies using a standardized data collection form. The collected data were then exchanged and reviewed. In case of discrepancies or disagreements, a third author (AR) was consulted. For the updated data extraction, JF took on the role of the third author in case no consensus could be reached. Data extraction was completed in November 2024. The following descriptive data was extracted from the studies: author name, publication year, country of study, language, study duration, study design, inclusion and exclusion criteria, randomization, risk of bias, study duration/follow-up-period, publication status, contact address, data analysis principle, success to close the perforation (yes/no). The following participant characteristics were extracted: intervention and comparison group size, median or mean age, sex, body mass index (kg/m^2^), concomitant diseases, ASA Score (1 to 5) [4], drinking history (yes/no), smoking history (yes/no), ulcer history (yes/no), use of Nonsteroidal Anti-Inflammatory Drugs (yes/no), APACHE II-Score (0–34 points), symptom duration (h), previous upper abdominal surgery. Characteristics of the interventions were extracted: open surgical (A) or laparoscopic (B), combined endoscopic and laparoscopic (C), combined endoscopic and radiological intervention (D), or conservative therapy (E). The following intraoperative findings were extracted: median size of perforation (mm), location of perforation (stomach (prepyloric, pyloric), duodenal), median blood loss (ml), success to close the perforation (yes/no), conversion to open treatment approach (yes/no).

The following predefined outcomes were extracted: mortality (in hospital, 30 days, 90 days) as main outcome, additional outcomes: morbidity (Clavien-Dindo-Classification) [5], operation time (minutes), postoperative length of hospital stay (days), postoperative pain (predefined in each study), leakage (all, blue dye test postoperative, contrast medium), nasogastric tube duration (days), time to resume diet (days), reoperation/reintervention (yes/no), decrease in CRP level and leukocyte count (before intervention to four days after intervention), perioperative analgesic requirement (number of patients), postoperative opiate use (days), cosmetic outcome (VAS score for scar appearance), total cost (Euro, USD), return to normal physical activity (days), intravenous fluid administration (days).

For each study, the risk of bias was assessed using the Cochrane Handbook for Systematic Reviews of Interventions and version 2 of the Cochrane “risk of bias” tool (RoB2) [6, 7].

The following characteristics were reviewed:

- Bias due to the randomization process.
- Bias due to deviations from the intended interventions.
- Bias due to missing outcome data.
- Bias in measurement of the outcome.
- Bias due to selection of the reported outcome.

The potential risk of bias was classified as either “low”, “some concerns” or “high”.

### Statistical analysis and data synthesis

This was an exploratory meta-analysis and an attempt to create a first overview of possible treatment options. Hence, summary statistics and pooled effects were reported though pooling to attain definitive evidence may not be advisable in this setting.

For all binary outcomes, treatment effects were expressed as ORs comparing an intervention and the corresponding control group and reported alongside a 95% CI. ORs for the pairwise meta-analyses were attained using Peto’s approach which is preferred for sparse event settings such as this one [8]. For the NMA the continuity correction (adding 0.5) was applied for study arms with zero events [9], since those served mostly illustrative purposes and are used to gather a general overview.

Continuous endpoints were analyzed as a mean difference alongside a 95% CI as summary measures.

To analyze the research question presented, a NMA was conducted to estimate treatment effects compared to the baseline effect of an open surgical control group. Due to the low occurrence of the interventions C, D, and E, the NMA was followed by a conventional pairwise meta-analysis assessing the effects of open (A) versus laparoscopic surgery (B).

The frequentist method based on graph theory for data synthesis was employed and the τ^2^ and I^2^ –statistics were used to assess between-trial variance [10]. Heterogeneity within designs and inconsistency were quantified by the Q statistic [11].

For the study by Lau et al. (1996), the arms A1 and A2 were condensed into a combined arm A, and B1 and B2 were combined into B, as our definition of the interventions did not distinguish between A1 and A2 or B1 and B2 [12]. In some of the trials, patients who were randomized into a laparoscopic group required switching to an open surgical treatment, marked as A/B in the extracted data. Patients who fell into that category were evaluated as patients from the laparoscopic group in accordance with the intention to treat principle (ITT). This might lead to a conservative estimate for the effect of laparoscopic intervention compared to open surgery when it comes to endpoints typically expected to be higher in the latter group (infections etc.).

### Missing data

If any data were missing, we reached out to the respective authors to request the necessary information.

Negm et al. compared the combined radiologic and endoscopic treatment approach with the surgical approach [14]. The surgical approach consisted of open and laparoscopic surgery. To improve the statistical calculation of the network, we solicited and received details regarding the number of participants in the open and laparoscopic groups, as well as data on gender distribution, mortality, postoperative complications (pneumonia, leakage, abdominal collection, renal failure, incisional hernia), length of hospital stay, and duration of surgery for each group. For this reason, this study was broken down into “A/B” (surgical group) as well as “A” (open surgical approach) and “B” (laparoscopic approach) in supplemental material 2. It was included in the pairwise analysis A versus B.

## Results

The initial search identified 1530 potentially relevant studies (Fig. 1). After checking the full texts, 16 studies were finally included in the network meta-analysis [12, 14–28]. The studies, originated from 8 countries and were published between 1989 and 2023. 1259 patients were included in these studies (A: 549, B: 521, A/B: 15, C: 13, D: 50, E: 40). We included four studies that were not indexed in the searched databases but were identified through other sources. Heterogeneity results for each analysis are shown in the corresponding figures in Supplemental Material 3.

Study characteristics, patient characteristics and outcomes are shown in Supplemental Material 2.

Publication biases were assessed using funnel plots, with findings reported through forest plots and summary of findings Table (13), which can be found in Supplemental Material 3.

**Fig. 3:**
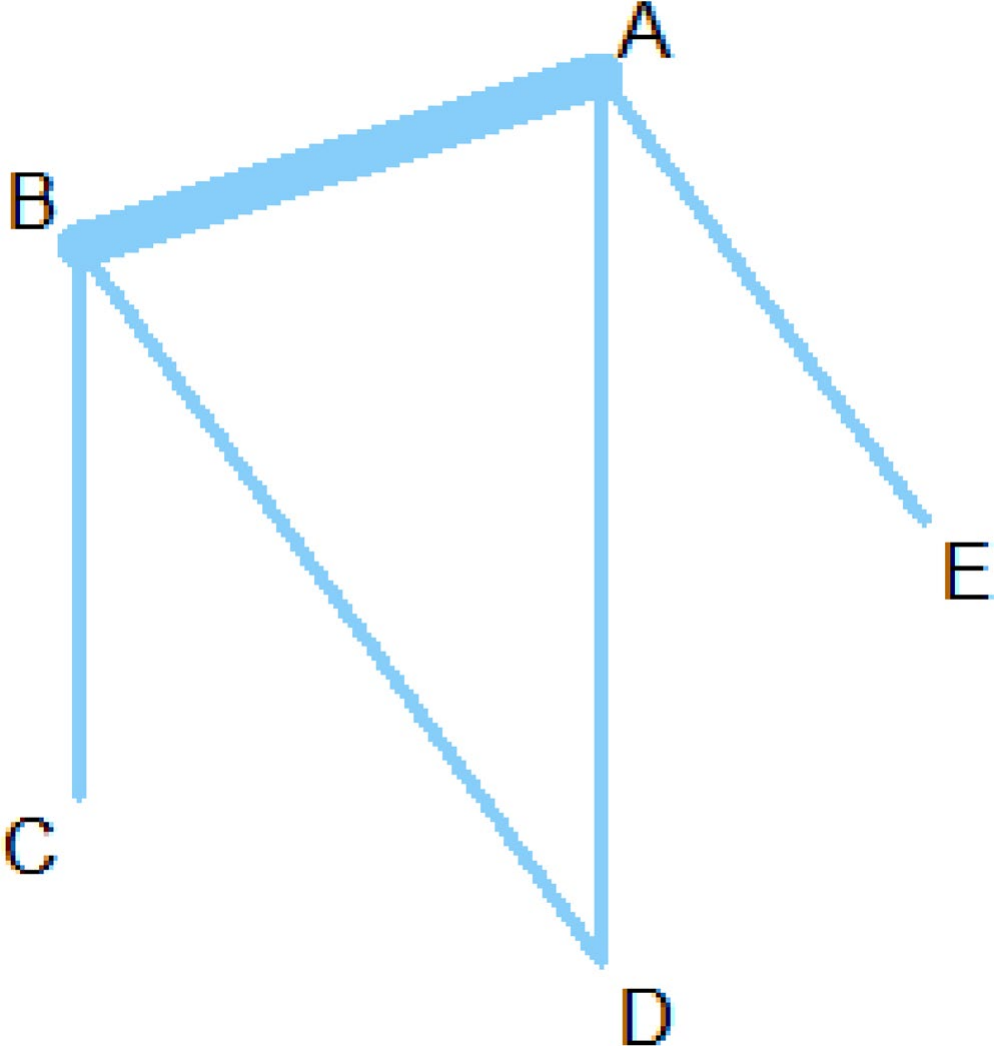
Net graph depicting comparisons for the primary endpoint mortality. **A** open surgical approach; **B** laparoscopic approach; **C** combined endoscopic - laparoscopic approach; **D** combined radiologic - endoscopic approach; **E** conservative approach

The network for the main outcome is shown in Fig. 3. The networks for the additional outcomes can be seen in Supplemental Material 3.

**Fig. 4:**
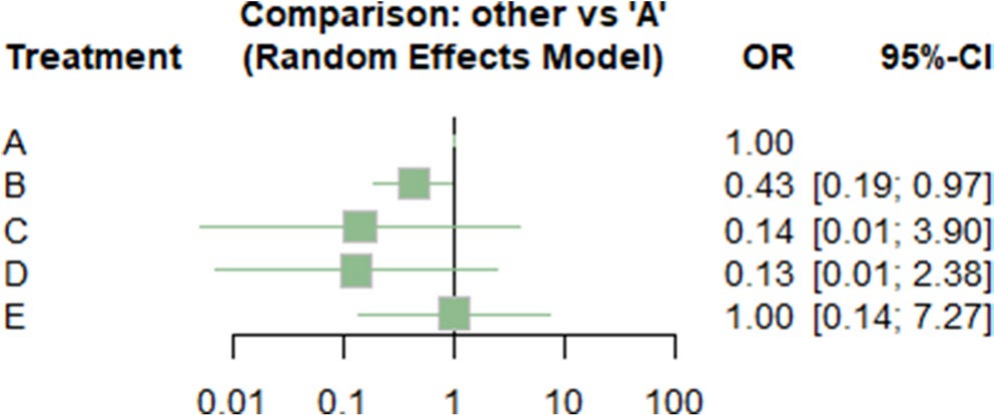
Forest plot of OR with 95% CI for mortality. **A** open surgical approach; **B** laparoscopic approach; **C** combined laparoscopic – endoscopic approach; CI, confidence interval; **D** combined endoscopic-radiologic approach; E, conservative approach; OR, odds ratio

In terms of the main outcome mortality, the laparoscopic approach significantly reduced mortality compared to open surgery (OR: 0.36; 95% CI 0.17 to 0.75; *p* = 0.0065), as shown in 14 studies (Table 1; Fig. 4). The combined laparoscopic-endoscopic and radiologic-endoscopic treatment approaches appear to favour lower mortality compared to open surgery. For the laparoscopic-endoscopic approach, the OR was 0.12 (95% CI 0.004–3.2, *p* = 0.20), while for the radiologic-endoscopic approach, the OR was 0.12 (95% CI 0.007–2.16, *p* = 0.15). However, these differences are not statistically significant (Table 1; Fig. 4).

When compared to open surgery, the conservative treatment approach shows no measurable difference in mortality, with an OR of 1.00 (95% CI 0.14–7.27, *p* = 1).

Only two studies assessed morbidity using the Clavien-Dindo classification. Consequently, we evaluated all reported postoperative complications. Due to limited data availability, statistical analysis could not be performed for complications such as fever, respiratory insufficiency, ARDS, cardiac issues, sepsis, fascia dehiscence, urinary tract infections, incisional hernia, cerebrovascular events, dysphagia, and abdominal collections. Detailed findings are provided in Supplemental Material 2.

**Table 1:** Network meta-analysis including OR with 95% CI and p-value for mortality

Treatment	OR	95%-CI	*p*-value
A	-	-	-
B	0.3596	[0.1720; 0.7515]	0.0065
C	0.1171	[0.0043; 3.2116]	0.2043
D	0.1192	[0.0066; 2.1640]	0.1504
E	1.0000	[0.1375; 7.2719]	1.0000

A, open surgical approach; B, laparoscopic approach; C, combined laparoscopic – endoscopic approach; CI, confidence interval; D, combined endoscopic-radiologic approach; E, conservative approach, OR, odds ratio; ORs, odds ratios

**Fig. 5:**
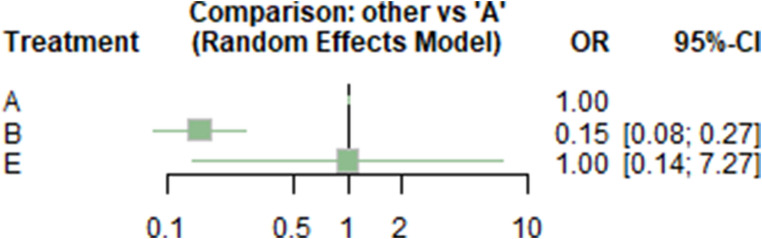
Forest plot of OR with 95% CI for wound infection

The laparoscopic approach is associated with significantly fewer wound infections compared to the open surgical approach (OR 0.15, 95% CI 0.08–0.27, *p* < 0.0001) (Table 2; Fig. 5).

**Table 2:** Network meta-analysis including OR with 95% CI and p-value for wound infection

Treatment	OR	95%-CI	*p*-value
A	-	-	-
B	0.1494	[0.0824; 0.2707]	< 0,0001
E	1.0000	[0.1375; 7.2719]	1.0000

A, open surgical approach; B, laparoscopic approach; C, combined laparoscopic – endoscopic approach; CI, confidence interval; D, combined endoscopic-radiologic approach; E, conservative approach, OR, odds ratio

No statistically significant differences were observed for the endpoints of hospital stay or postoperative complications, including leakage, pneumonia, abscess, ileus, hernia, organ failure, and chest complications (Supplemental Material 3). The combined radiologic- endoscopic treatment approach appears to favour lower rates of pneumonia (OR 0.09, 95% CI 0.005–1.84, *p* = 0.12), abscess (OR 0.09, 95% CI 0.003–2.55, *p* = 0.16), and hernia (OR 0.13, 95% CI 0.007–2.43, *p* = 0.17) (Supplemental Material 3). However, these findings were not statistically significant and were based on data from a single study. For the NMA, statistical analysis could not be performed for the endpoints “patients with complications” and ileus due to insufficient data availability.

Given that 14 RCTs compared the open with the laparoscopic approach, we conducted separate pairwise meta-analyses as sensitivity analysis. While the network was highly unstable, the pairwise meta-analysis showed consistent and robust results, with low heterogeneity across studies.

Comparison of both approaches revealed a significantly lower mortality with the laparoscopic approach (OR 0.36, 95% CI 0.17–0.75, *p* = 0.0065) (Supplemental Material 3).

The laparoscopic approach is associated with significantly fewer overall complications (OR 0.38, 95% CI 0.21–0.70) (Supplemental Material 3).

The laparoscopic approach is also associated with significantly lower probabilities of specific postoperative complications, including ileus (OR 0.33, 95% CI 0.18–0.59) and wound infection (OR 0.18, 95% CI 0.12–0.26), compared to the open surgical approach (Supplemental Material 3).

No statistically significant differences were observed for specific postoperative complications, including leakage, pneumonia, abscess, hernia, organ failure, and chest complications. Similarly, no statistically significant results were found for the endpoint of postoperative hospital stay (Supplemental Material 3).

Statistical analyses were not performed for several outcomes due to insufficient data availability, as fewer than three RCTs provided data for each. These outcomes include morbidity (Clavien-Dindo classification), postoperative complications (patients, fever, respiratory insufficiency, ARDS, cardiac problems, sepsis, fascia dehiscence, urinary tract infection, incisional hernia, cerebrovascular events, dysphagia, chest infections, abdominal collection), operation time, postoperative pain, nasogastric tube duration, time to resume diet, reoperation/reintervention, median blood loss, success in closing the perforation, conversion to open surgery, perioperative analgesic requirement, reduction in CRP levels and leukocyte count, postoperative opiate use, cosmetic outcomes, total cost, return to normal physical activity, and intravenous infusion administration. These outcomes are presented descriptively in the Supplemental Material 2.

### Bias analysis

For each study, the risk of bias was assessed using Cochrane Handbook for Systematic Reviews of Interventions and version 2 of the Cochrane “Risk of bias” tool (RoB2) [6, 7] (Supplemental Material 4).

Seven studies were classified as having “some concerns” regarding overall bias. Nine studies were classified as having “high risk of bias”.

For the domain “Randomization process” six studies where categorized with “some concerns” because critical patients were randomized to the less invasive treatment method, or there was no information on whether the allocation order was concealed until participants were enrolled and allocated to interventions [15, 20, 21, 24, 26, 27]. Two studies were categorized with “high risk of bias” because no random component, such as computer-generated numbers or sealed envelopes, was used [22, 28].

For the domain “Deviations from intended interventions” one study was categorized with “low risk of bias” [16]. Seven studies were categorized as “some concerns” because they did not specify their data analysis principle, although they described how they evaluated the participants who changed the intervention group or made a appropriate adjustment [15, 18, 22–24, 26, 29]. Eight studies were categorized with “high risk of bias” because they did not specify their data analysis and analyzed participants in the wrong intervention group or did not provide any information on whether analyzing participants in the wrong intervention group had a potential impact on the outcome [12, 14, 17, 19–21, 27, 28]. Bertleff et al. and Siu et al. explicitly reported analyzing their data according to the intention-to-treat (ITT) principle in their respective studies [16, 23]. Khedr et al. and Lau et al. (1998) reported their data potentially according to the as-treated and per protocol principles, respectively [19, 20]. The remaining studies did not specify their analytical methodology, leaving it unclear whether the data were analyzed based on the ITT, “as-treated” or “per-protocol” approach. Therefore, we applied the ITT principle, which is considered to be the gold standard, to the analysis of data from these other studies.

All studies were categorized with “low risk of bias” for the domain “missing outcome data” and domain “measurement of the outcome” except Saim et al. because they did not show statistical methods used to measure the outcomes [21]. For the domain “selection of reported result”, study by Ge et al. was categorized with “low risk of bias”, while all other studies were categorized with “some concerns”, as they did not publish a study protocol in advance [18].

## Discussion

The results of this network meta-analysis indicate that the laparoscopic approach significantly reduces mortality compared to open surgery and is associated with fewer wound infections and postoperative complications, making it a favorable option for managing PPU. These findings were further confirmed through pairwise comparisons, which validated the advantages of the laparoscopic approach over open surgery in reducing mortality and specific complications. In addition to these results, the combined radiologic and endoscopic approach appears to favor lower rates of postoperative complications, such as pneumonia, abscess, and hernia, compared to open surgery. However, these findings are based on data from a single study, limiting their reliability and generalizability. For other outcomes, such as leakage, hospital stay, and chest complications, no statistically significant differences were observed across the various treatment approaches.

The most recent meta-analysis comparing the laparoscopic approach with open surgery included nine studies with a total of 670 patients [30]. This analysis demonstrated the superiority of laparoscopic treatment over open surgery, showing lower rates of mortality (RR 0.37, 95% CI 0.15–0.90, *p* = 0.03), postoperative ileus (RR 0.43, 95% CI 0.20–0.95, *p* = 0.04), wound complications (RR 0.36, 95% CI 0.23–0.57, *p* < 0.0001), and a shorter hospital stay (MD −2.37, 95% CI −3.64 to −1.10, *p* = 0.0003). Our findings align with this meta-analysis regarding mortality, postoperative ileus, and wound complications. Moreover, our network meta-analysis expands on these results by incorporating alternative approaches and including additional studies comparing open surgery with laparoscopic techniques.

In another meta-analysis, Li et al. further demonstrated the advantages of laparoscopic treatment over open surgery in a comprehensive study comprising 29 studies with 17,228 patients, including four RCTs and 25 retrospective studies. Their results largely align with ours, reporting lower mortality (OR 0.36, 95% CI 0.27–0.49, *p* < 0.001), reduced rates of wound infections (OR 0.20, 95% CI 0.17–0.24, *p* < 0.001), and pneumonia (OR 0.59, 95% CI 0.41–0.87, *p* = 0.01), as well as shorter hospital stays and less blood loss with the laparoscopic approach [31]. Notably, our study identified significant benefits regarding postoperative ileus, which was not reported as significant in the analysis by Li et al., highlighting an additional advantage of the laparoscopic approach.

Currently, there are no meta-analyses or systematic reviews that directly compare alternative or conservative treatment approaches with each other or with open surgery. This highlights a critical gap in the literature and underscores the need for further high-quality RCTs to evaluate these alternative strategies comprehensively. The conservative approach according to the Taylor regimen consists of treatment with nasogastric suction, intravenous administration of antibiotics, intravenous administration of analgesics, adequate hydration, electrolyte balancing, intravenous administration of H2 receptor blockers or PPI [17, 32]. There is ongoing discussion in the literature regarding the effectiveness of this approach. Studies have shown that patients can achieve acceptable results in terms of mortality and morbidity with the conservative method [33–37]. We could not demonstrate any statistically significant results regarding the conservative management.

The combined approach of laparoscopy and endoscopy is scarcely represented in the literature. A few older case series and studies described this combination as promising [38–40]. For instance, Bergström et al. reported the use of self-expanding metal stents alongside drainage, suggesting that this combination, along with laparoscopic diagnosis, could be an alternative to surgery, particularly for ulcers that are difficult to close or in patients with severe comorbidities [40]. However, our study did not yield statistically significant results, likely reflecting the limited evidence supporting the success of this approach.

Regarding the combination of laparoscopy and radiological intervention, no other studies could be identified in the current literature apart from the RCT included in our analysis [5]. As discussed, this approach may offer advantages in terms of mortality and morbidity, but these findings require validation through additional high-quality RCTs.

Robot-assisted surgery is increasingly utilized in clinical practice. Studies highlight advantages such as three-dimensional visualization and greater freedom of movement [41, 42]. In the treatment of perforated ulcers, robot-assisted procedures may provide benefits compared to laparoscopy, including reduced blood loss, lower complication rates, and shorter hospital stay. However, the available evidence is limited, its application in emergency general surgery is still uncommon, preparation and operation times and thus time to definite closure of the PPU might be longer, and higher costs remain a significant barrier [43–45].

This review is the first one summarizing the entire evidence regarding approaches for the treatment of PPU. As such, it provides a valuable insight in an understudied field and presents a useful overview for the current state of the art with regard to multiple clinically relevant outcomes The strength of this study lies in its comprehensive inclusion of all relevant RCTs, offering more focused insights into alternative treatment approaches. To date, only meta-analyses and systematic reviews comparing laparoscopic and open surgical approaches had been conducted. No NMA had yet incorporated all currently available RCTs to provide a comprehensive evaluation of different treatment approaches for perforated peptic ulcers. This base of evidence is a good starting point to motivate further clinical trials addressing the efficacy of treatments combined endoscopic-laparoscopic, combined radiologic-endoscopic, and conservative approach.

The study landscape with regard to approaches for the treatment of PPU is characterized by a high degree of heterogeneity which made pooling effects and estimating overall treatment efficacy more unreliable. The networks also suffered from an imbalance, with most available comparisons being between open and laparoscopic surgery. There was one study of each of the other treatment approaches. Therefore, these statements could not be supported by a large number of studies, and the treatment effects for these approaches remained imprecise and unreliable due to the limited evidence. Additionally, a high risk of bias was identified in this analysis, necessitating caution when interpreting and applying the findings. Moreover, many older studies were included, conducted at a time when standards for medical research were not yet well established. It is also possible that different clinical standards applied back then than today.

As a consequence, pairwise meta-analyses between open and laparoscopic surgery were conducted as sensitivity analyses, as well as treatment comparison of major interest was added.

No other comparisons were performed in the pairwise analysis. These pairwise meta-analyses demonstrated high stability and low heterogeneity, reinforcing the reliability of the observed treatment effects. Moreover, the two treatments in question had some flow of patients between them, as it is standard practice to resort to open surgery should the laparoscopic approach prove unfeasible mid-treatment. Since we conducted all analyses according to the ITT principle, some patients were counted towards laparoscopic intervention in reality ended up having open surgery. This might have led to a rather conservative effect estimate of the laparoscopic treatment versus the open surgical approach, which was tolerable since a more conservative estimate is preferable to a biased one towards a more extreme treatment effect.

Another limitation comes through the relative rarity of some of the events in question. We tried to account for this problem by using methods that have proven to be robust in rare events settings, yet sparse data must always be considered a limiting factor in meta-analysis. The funnel plots for each outcome either appear unremarkable or are not conclusive due to the small number of studies. They should serve as orientation when assessing robustness of results.

Despite all these challenges and issues regarding the data in question we are confident that this review has merit as an exploratory insight into the presented field. Whilst overall treatment effects presented here should not be used as the sole argument for treatment decisions they do serve as a good overview and might aid decision-making in the face of uncertainty.

## Conclusions

This network meta-analysis demonstrates that the laparoscopic approach is associated with lower mortality, fewer postoperative complications overall, and reduced rates of specific complications such as wound infections and ileus compared to the open surgical approach. There appears to be a suggestion that the combined endoscopic and radiologic approach favors lower risks of mortality and complications such as pneumonia, abscess, and hernia compared to open surgery. However, the evidence is insufficient to draw definitive conclusions, and further RCTs are required. The benefits of the combined laparoscopic-endoscopic approaches remain unclear, emphasizing the need for further research to determine their specific indications and potential advantages.

## Supplementary Information

Below is the link to the electronic supplementary material.

Supplementary file 1 (44.0KB)

Supplementary file 2 (120KB)

Supplementary file 3 (228KB)

Supplementary file 4 (640KB)

## Data Availability

Yes, I have research data to declare. The data used in the manuscript consist of both self-generated data and data extracted from previously published articles. Where applicable, the relevant sources and references are provided in the manuscript. The data underlying the findings of this study are not publicly accessible due to privacy constraints but can be provided upon reasonable request to the corresponding author.
