# Supplementary material for: Approaches for the treatment of perforated peptic ulcers: a network meta-analysis of randomized controlled trials
Source: Langenbecks Arch Surg. 2025 Sep 5;410(1):266. doi: 10.1007/s00423-025-03848-9 (PMC12413338; doi:10.1007/s00423-025-03848-9)
Supplement: Supplementary file 1 — (44.0KB) [file 423_2025_3848_MOESM1_ESM.docx]

**Main aspects**

### P

|  | perforated peptic ulcers |  |
| --- | --- | --- |

### I

|  | Treatment |  |
| --- | --- | --- |

## Search guide overview

| 1 | P |  |
| --- | --- | --- |
| 2 | I |  |
| 3 | Studies | If needed |

## Databases and platforms involved

- PubMed (via NCBI)
- EMBASE (via Elsevier)
- Cochrane Library (via Wiley)
- Cinahl (via Ebsco)
- ClinicalTrials.Gov (via [www.clinicaltrials.gov](http://www.clinicaltrials.gov))
- ICTRP (via <https://trialsearch.who.int/Default.aspx>)

## PubMed

### P

| 1 | **"Peptic Ulcer Perforation"[Mesh] OR**  (Peptic[tiab] AND  Ulcer[tiab] AND  Perforat*[tiab]) |
| --- | --- |

### I

| 2 | Omentum*[tiab] OR  Omental*[tiab] OR  graham*[tiab] OR  "Cellan Jones"[tiab] OR  Cellanjones[tiab] OR  "Falciform Ligament*"[tiab] OR  "Over the scope*"[tiab] OR  "Metal stent*"[tiab] OR |
| --- | --- |
|  | **"General Surgery"[Mesh] OR**  **"Surgical Procedures, Operative"[Mesh] OR**  **"surgery" [Subheading] OR**  Operat*[tiab] OR  Surg*[tiab] OR  Excision*[tiab] OR  Dissection*[tiab] OR  resect*[tiab] OR  removal*[tiab] OR  ectomy[tiab] OR  ectomies[tiab] OR  Preoperat*[tiab] OR  Postoperat*[tiab] OR  Perioperat*[tiab] OR |
|  | **"Endoscopes"[Mesh:NoExp] OR**  **"Radiology, Interventional"[Mesh] OR**  **"Laparoscopes"[Mesh] OR**  **"Minimally Invasive Surgical Procedures"[Mesh] OR**  "Interventional Radio*"[tiab] OR  "Minimally invasive repair*"[tiab] OR  "Minimal invasive repair*"[tiab] OR  Laparoscop*[tiab] OR  Endoscop*[tiab] OR  Celioscop*[tiab] OR  Peritoneoscop*[tiab] |

### Studies

| 3 | randomized controlled trial[pt] OR  "Randomized Controlled Trials as Topic"[Mesh] OR controlled clinical trial[pt] OR  randomized[tiab] OR  placebo[tiab] OR  drug therapy[sh] OR  randomly[tiab] OR  trial[tiab] OR  groups[tiab] OR  study[tiab] OR  studies[tiab] |
| --- | --- |

### Strings

| 1 | "Peptic Ulcer Perforation"[MeSH Terms] OR ("Peptic"[Title/Abstract] AND "Ulcer"[Title/Abstract] AND "perforat*"[Title/Abstract]) |
| --- | --- |
| 2 | "omentum*"[Title/Abstract] OR "omental*"[Title/Abstract] OR "graham*"[Title/Abstract] OR "Cellan Jones"[Title/Abstract] OR "Cellanjones"[Title/Abstract] OR "falciform ligament*"[Title/Abstract] OR "over the scope*"[Title/Abstract] OR "metal stent*"[Title/Abstract] OR "General Surgery"[MeSH Terms] OR "surgical procedures, operative"[MeSH Terms] OR "surgery"[MeSH Subheading] OR "operat*"[Title/Abstract] OR "surg*"[Title/Abstract] OR "excision*"[Title/Abstract] OR "dissection*"[Title/Abstract] OR "resect*"[Title/Abstract] OR "removal*"[Title/Abstract] OR "ectomy"[Title/Abstract] OR "ectomies"[Title/Abstract] OR "preoperat*"[Title/Abstract] OR "postoperat*"[Title/Abstract] OR "perioperat*"[Title/Abstract] OR "Endoscopes"[MeSH Terms:noexp] OR "radiology, interventional"[MeSH Terms] OR "Laparoscopes"[MeSH Terms] OR "Minimally Invasive Surgical Procedures"[MeSH Terms] OR "interventional radio*"[Title/Abstract] OR "minimally invasive repair*"[Title/Abstract] OR "minimal invasive repair*"[Title/Abstract] OR "laparoscop*"[Title/Abstract] OR "endoscop*"[Title/Abstract] OR "celioscop*"[Title/Abstract] OR "peritoneoscop*"[Title/Abstract] |
| 3 | "randomized controlled trial"[Publication Type] OR "Randomized Controlled Trials as Topic"[MeSH Terms] OR "controlled clinical trial"[Publication Type] OR "randomized"[Title/Abstract] OR "placebo"[Title/Abstract] OR "drug therapy"[MeSH Subheading] OR "randomly"[Title/Abstract] OR "trial"[Title/Abstract] OR "groups"[Title/Abstract] OR "study"[Title/Abstract] OR "studies"[Title/Abstract] |
| 4 | #1 AND #2 AND #3 |

## Embase

### P

| 1 | **'ulcer perforation'/exp OR**  (Peptic NEAR/4 Ulcer NEAR/4 Perforat*):ti,ab,kw |
| --- | --- |

### I

| 2 | Omentum*:ti,ab,kw OR  Omental*:ti,ab,kw OR  graham*:ti,ab,kw OR  "Cellan Jones":ti,ab,kw OR  Cellanjones:ti,ab,kw OR  "Falciform Ligament*":ti,ab,kw OR  "Over the scope*":ti,ab,kw OR  "Metal stent*":ti,ab,kw OR |
| --- | --- |
|  | **'surgery'/exp OR**  Operat*:ti,ab,kw OR  Surg*:ti,ab,kw OR  Excision*:ti,ab,kw OR  Dissection*:ti,ab,kw OR  resect*:ti,ab,kw OR  removal*:ti,ab,kw OR  ectomy:ti,ab,kw OR  ectomies:ti,ab,kw OR  Preoperat*:ti,ab,kw OR  Postoperat*:ti,ab,kw OR  Perioperat*:ti,ab,kw OR |
|  | **'endoscope'/de OR**  **'interventional radiology'/exp OR**  **'laparoscope'/exp OR**  **'minimally invasive surgery'/exp OR**  "Interventional Radio*":ti,ab,kw OR  "Minimal* invasive repair*":ti,ab,kw OR  Laparoscop*:ti,ab,kw OR  Endoscop*:ti,ab,kw OR  Celioscop*:ti,ab,kw OR  Peritoneoscop*:ti,ab,kw |

### Studies

Box 3.e Cochrane Highly Sensitive Search Strategy for identifying controlled trials in Embase: (2020 revision); Embase.com format. S. 63-64. The search term "study" was added to make the search more sensitive

<https://training.cochrane.org/handbook/current/chapter-04-technical-supplement-searching-and-selecting-studies>

| 3 | (‘randomized controlled trial’/de OR  ‘controlled clinical trial’/de OR  random*:ti,ab,tt OR  ‘randomization’/de OR  ‘intermethod comparison’/de OR  placebo:ti,ab,tt OR  (compare:ti,tt OR compared:ti,tt OR comparison:ti,tt)  OR  ((evaluated:ab OR evaluate:ab OR evaluating:ab OR assessed:ab OR assess:ab) AND (compare:ab OR compared:ab OR comparing:ab OR comparison:ab))  OR  (open NEXT/1 label):ti,ab,tt OR  ((double OR single OR doubly OR singly) NEXT/1 (blind OR blinded OR blindly)):ti,ab,tt OR  ‘double blind procedure’/de OR  (parallel NEXT/1 group*):ti,ab,tt OR  (crossover:ti,ab,tt OR ‘cross over’:ti,ab,tt)  OR  ((assign* OR match OR matched OR allocation) NEAR/6 (alternate OR group OR groups OR intervention OR interventions OR patient OR patients OR subject OR subjects OR participant OR participants)):ti,ab,tt  OR  (assigned:ti,ab,tt OR allocated:ti,ab,tt)  OR  (controlled NEAR/8 (study OR design OR trial)):ti,ab,tt  OR  (volunteer:ti,ab,tt OR volunteers:ti,ab,tt) OR  ‘human experiment’/de OR  trial:ti,tt)  OR  study:ti,ab,kw |
| --- | --- |

### Strings

| 1 | 'ulcer perforation'/exp OR ((peptic NEAR/4 ulcer NEAR/4 perforat*):ti,ab,kw) |
| --- | --- |
| 2 | omentum*:ti,ab,kw OR omental*:ti,ab,kw OR graham*:ti,ab,kw OR 'cellan jones':ti,ab,kw OR cellanjones:ti,ab,kw OR 'falciform ligament*':ti,ab,kw OR 'over the scope*':ti,ab,kw OR 'metal stent*':ti,ab,kw OR 'surgery'/exp OR operat*:ti,ab,kw OR surg*:ti,ab,kw OR excision*:ti,ab,kw OR dissection*:ti,ab,kw OR resect*:ti,ab,kw OR removal*:ti,ab,kw OR ectomy:ti,ab,kw OR ectomies:ti,ab,kw OR preoperat*:ti,ab,kw OR postoperat*:ti,ab,kw OR perioperat*:ti,ab,kw OR 'endoscope'/de OR 'interventional radiology'/exp OR 'laparoscope'/exp OR 'minimally invasive surgery'/exp OR 'interventional radio*':ti,ab,kw OR 'minimally invasive repair*':ti,ab,kw OR 'minimal invasive repair*':ti,ab,kw OR laparoscop*:ti,ab,kw OR endoscop*:ti,ab,kw OR celioscop*:ti,ab,kw OR peritoneoscop*:ti,ab,kw |
| 3 | 'randomized controlled trial'/de OR 'controlled clinical trial'/de OR random*:ti,ab,tt OR 'randomization'/de OR 'intermethod comparison'/de OR placebo:ti,ab,tt OR compare:ti,tt OR compared:ti,tt OR comparison:ti,tt OR ((evaluated:ab OR evaluate:ab OR evaluating:ab OR assessed:ab OR assess:ab) AND (compare:ab OR compared:ab OR comparing:ab OR comparison:ab)) OR ((open NEXT/1 label):ti,ab,tt) OR (((double OR single OR doubly OR singly) NEXT/1 (blind OR blinded OR blindly)):ti,ab,tt) OR 'double blind procedure'/de OR ((parallel NEXT/1 group*):ti,ab,tt) OR crossover:ti,ab,tt OR 'cross over':ti,ab,tt OR (((assign* OR match OR matched OR allocation) NEAR/6 (alternate OR group OR groups OR intervention OR interventions OR patient OR patients OR subject OR subjects OR participant OR participants)):ti,ab,tt) OR assigned:ti,ab,tt OR allocated:ti,ab,tt OR ((controlled NEAR/8 (study OR design OR trial)):ti,ab,tt) OR volunteer:ti,ab,tt OR volunteers:ti,ab,tt OR 'human experiment'/de OR trial:ti,tt OR study:ti,ab,kw |
| 4 | #1 AND #2 AND #3 |

**To switch off PubMed**

| 5 | #4 NOT ([medline]/lim OR [pubmed-not-medline]/lim) |
| --- | --- |

**To exclude document types not of interest**

| 6 | #5 NOT ('Conference Abstract'/it OR 'Note'/it) |
| --- | --- |

## Cochrane Library

### P

| 1 | **[mh "Peptic Ulcer Perforation"] OR**  Peptic NEAR/3 Ulcer NEAR/3 Perforat*:ti,ab,kw |
| --- | --- |

### I

| 2 | Omentum*:ti,ab,kw OR  Omental*:ti,ab,kw OR  graham*:ti,ab,kw OR  "Cellan Jones":ti,ab,kw OR  Cellanjones:ti,ab,kw OR  Falciform NEAR/3 Ligament*:ti,ab,kw OR  Over NEAR/3 the NEAR/3 scope*:ti,ab,kw OR  Metal NEAR/3 stent*:ti,ab,kw OR |
| --- | --- |
|  | **[mh "General Surgery"] OR**  **[mh "Surgical Procedures, Operative"] OR**  Operat*:ti,ab,kw OR  Surg*:ti,ab,kw OR  Excision*:ti,ab,kw OR  Dissection*:ti,ab,kw OR  resect*:ti,ab,kw OR  removal*:ti,ab,kw OR  ectomy:ti,ab,kw OR  ectomies:ti,ab,kw OR  Preoperat*:ti,ab,kw OR  Postoperat*:ti,ab,kw OR  Perioperat*:ti,ab,kw OR |
|  | **[mh ^"Endoscopes"] OR**  **[mh "Radiology, Interventional"] OR**  **[mh "Laparoscopes"] OR**  **[mh "Minimally Invasive Surgical Procedures"] OR**  Interventional NEAR/3 Radio*:ti,ab,kw OR  Minimal* NEAR/3 invasive NEAR/3 repair*:ti,ab,kw OR  Laparoscop*:ti,ab,kw OR  Endoscop*:ti,ab,kw OR  Celioscop*:ti,ab,kw OR  Peritoneoscop*:ti,ab,kw |

### Strings

1-2 as in the tables above

| 3 | #1 AND #2 |
| --- | --- |

## CINAHL

### P

| 1 | **MH "Peptic Ulcer Perforation" OR**  TX (Peptic N3 Ulcer N3 Perforat*) |
| --- | --- |

### I

| 2 | **(MH "Surgery, Operative+" OR**  **MH "Endoscopes" OR**  **MH "Laparoscopy" OR**  (TX (Omentum* OR  Omental* OR  graham* OR  "Cellan Jones" OR  Cellanjones OR  Falciform N3 Ligament* OR  Over N3 the N3 scope* OR  Metal N3 stent* OR |
| --- | --- |
|  | Operat* OR  Surg* OR  Excision* OR  Dissection* OR  resect* OR  removal* OR  ectomy OR  ectomies OR  Preoperat* OR  Postoperat* OR  Perioperat* OR |
|  | Interventional N3 Radio* OR  Minimal* N3 invasive N3 repair* OR  Laparoscop* OR  Endoscop* OR  Celioscop* OR  Peritoneoscop*))) |

### Strings (due to the few hits the aspect studies is not involved)

| 1 | MH "Peptic Ulcer Perforation" OR TX (Peptic N3 Ulcer N3 Perforat*) |
| --- | --- |
| 2 | (MH "Surgery, Operative+" OR MH "Endoscopes" OR MH "Laparoscopy" OR (TX (Omentum* OR Omental* OR graham* OR "Cellan Jones" OR Cellanjones OR Falciform N3 Ligament* OR Over N3 the N3 scope* OR Metal N3 stent* OR Operat* OR Surg* OR Excision* OR Dissection* OR resect* OR removal* OR ectomy OR ectomies OR Preoperat* OR Postoperat* OR Perioperat* OR Interventional N3 Radio* OR Minimal* N3 invasive N3 repair* OR Laparoscop* OR Endoscop* OR Celioscop* OR Peritoneoscop*))) |
| 3 | #1 AND #2 |

## ClinicalTrial.gov

<http://www.clinicaltrials.gov/>

### P

| 1 | "Peptic Ulcer Perforation" OR  "Peptic Ulcer Perforations" |
| --- | --- |

### I

| 2 | Omentum OR  Omental OR  graham OR  "Cellan Jones" OR  Cellanjones OR  "Falciform Ligament" OR  "Over the scope" OR  "Metal stent" OR |
| --- | --- |
|  | Operation OR  Surgery OR  Excision OR  Dissection OR  resection OR  removal OR  ectomy OR  ectomies OR  Preoperation OR  Postoperation OR  Perioperation OR |
|  | "Interventional Radio" OR  "Minimally invasive repair" OR  "Minimal invasive repair" OR  Laparoscopy OR  Endoscopy OR  Celioscopy OR  Peritoneoscopy |

### Strings

| 1 | (EXPAND[Concept] ( "Peptic Ulcer Perforation" OR "Peptic Ulcer Perforations" )) |
| --- | --- |
| 2 | AND  (Omentum OR Omental OR graham OR EXPAND[Concept] "Cellan Jones" OR Cellanjones OR EXPAND[Concept] "Falciform Ligament" OR EXPAND[Concept] "Over the scope" OR EXPAND[Concept] "Metal stent" OR Operation OR Surgery OR Excision OR Dissection OR resection OR removal OR ectomy OR ectomies OR Preoperation OR Postoperation OR Perioperation OR EXPAND[Concept] "Interventional Radio" OR EXPAND[Concept] "Minimally invasive repair" OR EXPAND[Concept] "Minimal invasive repair" OR Laparoscopy OR Endoscopy OR Celioscopy OR Peritoneoscopy) |

| 3 | 1 AND 2 |
| --- | --- |

## International Clinical Trials Registry Platform ICTRP (WHO Trials)

<https://trialsearch.who.int/> (simple)

<https://trialsearch.who.int/AdvSearch.aspx> (advanced)

### P

| 1 | Peptic Ulcer Perforation |
| --- | --- |

### I

| 2 | Omentum OR  Omental OR  graham OR  Cellan Jones OR  Cellanjones OR  Falciform Ligament OR  Over the scope OR  Metal stent OR |
| --- | --- |
|  | Surgery OR  Interventional Radio OR  Minimally invasive repair OR  Minimal invasive repair OR  Laparoscopy OR  Endoscopy OR  Celioscopy OR  Peritoneoscopy |

### Strings (in advanced mode)

| **Fields** | **String** |
| --- | --- |
| 1 (Condition) | Peptic Ulcer Perforation |
| 2 (Intervention) | Omentum OR Omental OR graham OR Cellan Jones OR Cellanjones OR Falciform Ligament OR Over the scope OR Metal stent OR Surgery OR Interventional Radio OR Minimally invasive repair OR Minimal invasive repair OR Laparoscopy OR Endoscopy OR Celioscopy OR Peritoneoscopy |

| 3 | 1 AND 2 |
| --- | --- |

### Strings (in simple mode)

| 1 | Peptic Ulcer Perforation AND surgery |
| --- | --- |
