# Supplementary material for: Approaches for the treatment of perforated peptic ulcers: a network meta-analysis of randomized controlled trials
Source: Langenbecks Arch Surg. 2025 Sep 5;410(1):266. doi: 10.1007/s00423-025-03848-9 (PMC12413338; doi:10.1007/s00423-025-03848-9)
Supplement: Supplementary file 2 — (120KB) [file 423_2025_3848_MOESM2_ESM.docx]

*Descriptive data from the included studies*

| Study (author name, publication year) | Country | Language | Study duration | Study design | Follow-Up period (months |
| --- | --- | --- | --- | --- | --- |
| Crofts et al.  1989 | Great Britain | Englisch | 11/1985- 12/1986 | prospective randomized controlled trial | 6 |
| Lau et al.  1996 | China | English | 08/1992-12/1994 | prospective randomzed trial, single center | 1 |
| Lau et al.  1998 | China | English | 09/1995 - 07/1996 | prospective randomized trial | N/A |
| Siu et al.  2002 | China | English | 01/1994 - 06/1997 | prospective randomized trial | 1, 3, 6 |
| Bertleff et al.  2009 | Netherlands | Englisch | 03/1999- 07/2005 | multicenter randomized controlled trial | 1, 6, 12 |
| Shah et al.  2015 | India | English | 01/2009 - 07/2011 | unicentric, nonblinded, prospective, randomized study | N/A |
| Zedan et al.  2015 | Egypt | English | 10/2012- 04/2014 | RCT | 1,5 |
| Zhang, Wang  2015 | China | Standard chinese | 08/2013 - 08/2014 | prospective randomized trial | N/A |
| Ge et al.  2016 | China | English | 01/2010 - 06/2014 | prospective, singlecenter, randomized, controlled, nonblinded clinical trial | 10 days; 1, 3 |
| Abdullah et al.  2018 | Egypt | English | 12/2013 - 12/2017 | RCT | N/A |
| Srivastava et al.  2018 | India | English | 04/2012 - 04/2013 | RCT | N/A |
| Arroyo Vázquez et al.  2020 | Sweden | English | 12/2014 - 08/2018 | multicenter randomized controlled trial | none |
| Negm et al.  2022 | Egypt | English | 12/2019 - 08/2021 | prospective randomized controlled clinical trial | 3 |
| Khedr et al.  2023 | Egypt | English | 05/2017 -10/2021 | two-center, prospective, randomized | N/A |
| Saim et al.  2023 | Pakistan | English | 01/2022 12/2022 | prospective comparative study | N/A |
| Saleem et al.  2023 | Egypt | English | 04/2022 - 02/2023 | RCT | N/A |

N/A: not available, RCT: randomized controlled trial.

| Study (Author name  Publication year) | inclision criteria | exclusion criteria | Randomization |
| --- | --- | --- | --- |
| Crofts et al.  1989 | clinical diagnosis of PPU; diagnosis based on history of sudden onset of epigastric pain associated with typical boardlike rigidity of the upper abdomen; informed consent was obtained from each patient before therapy | N/A | opening numbered, opaque, sealed envelope containing the treatment option |
| Lau et al.  1996 | all patients admitted with a clinical diagnosis of perforated peptic ulcer were considered part of the study. The absence of free gas under the diaphragm on plain radiography did not exclude patients from this study. | 1) complicated ulcers that required definitive ulcer surgery; 2) associated bleeding ulcers; 3) unsuitability for laparoscopic procedures like previous operations; 4) serious associated cardiopulmonary diseases that precluded a long operation; 5) no consent from patient for randomization; and 6) clinical sealed-off perforated ulcers. | Computer-generated blocked random numbers were used to assign the type of surgery, which was written on a card sealed in a completely opaque envelope. Envelopes were drawn randomly by the senior duty nurse in the operating department when an operating room was booked after the clinical decision to operate had been made. |
| Lau et al.  1998 | Age >16 & <70 years; diagnosis of PPU | concurrent ulcer bleeding mandating definitive surgery; immunisupression, recent steroid use, chemotherapy, or radiotherapy | not further specified |
| Siu et al.  2002 | diagnosis PPU; older than 16 years | surgical diagnosis other than PPU | randomization performed after decision was made for surgery; by person not otherwise involved in clinical setting; consecutively numbered opaque sealed envelopes; assigned by computer-generated random numbers |
| Bertleff et al.  2009 | patients with symptoms of the clinical diagnosis of PPU | inability to read the Dutch language patient information booklet, inability to complete informed consent, prior upper abdominal surgery, and current pregnancy. | Surgeons contacted the study coordinator after the patients had provided informed consent and randomization took place by opening a sealed envelope. The envelope randomization was based on a computer-generated list provided by the trial statistician. |
| Shah et al.  2015 | N/A | N/A | randomly assigned to group A or B |
| Zedan et al.  2015 | "patients with suspected perforated duodenal ulcer based on clinial assessment, investigation, and confirmed by exploration, either male or female of any age with Boey's score 0 or 1" | "patients with Boey' score 2 or 3, gastric outlet obstruction, bleeding ulcer, and previous abdominal exploration" | "patients were divided by random serial number method into two groups" |
| Zhang, Wang  2015 | Fulfill the relevant diagnostic criteria for PPU, typical clinical manifestation of PPU, confirmed by routine endoscopy or radiological examination, presence of severe infections, inflammation & edema in the abdominal cavity, and a large amount of purulent exudate present. | Combination with malignant diseases, individuals with tumors or tumor metastases, individuals with complications such as bleeding & obstructions, individuals with other systemic organic diseases who cannot undergo perforation repair; individuals who cannot cooperate. | N/A |
| Ge et al.  2016 | consecutive adult patients with a clinical diagnosis of PPU | (1) refusal to receive surgery; (2) serious cardiopulmonary dysfunction; (3) peptic ulcers that were simultaneously bleeding and perforated; (4) suspected perforation of gastric cancer; and (5) current pregnancy | “patients were allocated randomly in a 1:1 ratio to the OR group or LR group, in accordance with a randomization sequence that was computer-generated in the coordinating office and was informed to doctors by telephone after general anesthesia was delivered to the patient.” |
| Abdullah et al.  2018 | 16-70 years, provisional diagnosis PPU (general peritonitis and pneumooeritoneum) | "Patients with delayed presentation (>48h), absolute contraindication for laparoscopy (…), malignant ulcers (….), rare sites of peptic ulcer (….), and other complications with perforated peptic ulcer" | "all included patients were randomly divided into tow groups (…), who were managed woth laparoscopic and open omental patch repair", "randomization was performed by a computer-generated schedule, and the results were sealed into envelopes. The envelopes were drawn and opened in the operating room by a nurse not otherwise involved in the study |
| Arroyo Vázquez et al.  2020 | all patients at ER with abdominal pain, clinical signs of perforation of upper GIT, free abdominal air on CT | non-surgical candidates or patients in critical condition unable to sign; patients under 18, patients in need of translator | Randomization was done by allocation of patients in a 1:1 ratio in balanced blocks. Envelopes were prepared with slips of paper marked either stent oder surgery group |
| Negm et al.  2022 | patients >18 - <60 years with acute perforated gastric or duodenal ulcers, with early chemical peritonitis and no septic shock | patientes <18, >60 years, in severe septic shock or presence of massive amount of intraperitoneal, subhepatic, perisplenic and pelvic free fluid; size of perforation >30mm in diameter | drawing of sealed envelopes containing computer-generated random numbers prepared by a third party before the start of the intervention |
| Khedr et al.  2023 | acute abdomen with PPU diagnosed clinically within 48 h of the onset of symptoms; patient age between 16-70 years | patients with absolute contraindications for laparoscopy; those with malignant ulcers, pregnant woman with PPU | sealed envelpoes; randomly selected according to a computer-generated schedule |
| Saim et al.  2023 | all patients with diagnosis of a PPI, regardless of age or gender were included | patients with sealed perforations were omitted; patients with radiological sign of gas under the diaphragm but no clinical signs of peritonitis, patients with shcok who dont respond to intravenous fluids & vasopressor medication + RR <90 mmHg, with perforations other nen PPU | randomly devided into to groups by lottery method |
| Saleem et al.  2023 | "1.patients who agreed to participate in the study; 2 patients with perforated peptic ulcer based on clinical assessment, investigations, and confirmed by exploration; 3. either male or female of any age" | "1. patients with a surgical diagnosis other than a perforated peptic ulcer; 2. patients with gastric outlet obstruction.; 3. bleeding ulcer; 4. previous abdominal exploration that results in upper abdominal scare, e.g. midline, paramedian, transverse epigastric incisions, etc.; 5. patients who absconded or left the study or died during the period of study; 6. patients with cardiac and chest conditions (excluded from laparoscopic)" | "patients will be divided by the random serial number method into two groups |

A: open surgical approach, B: laparoscopic approach, N/A: not available, PPU: perforated peptic ulcer.

*Patient characteristics from the included studies*

| Study | intervention/ treatment approach | Size of intervention | Average age (years) +/- SD | Median age (Range) (years) | Gender (women / men) | Median Body Mass Index (kg/m^2^) (range) |
| --- | --- | --- | --- | --- | --- | --- |
| Crofts et al.  1989 | E | 40 | 55,4  +/-16 | N/A | 6/34 | N/A |
|  | A | 43 | 53,7  +/- 21 | N/A | 8/35 | N/A |
| Lau et al.  1996 | A1 | 21 | 48 | N/A | 4/17 | N/A |
|  | A2 | 24 | 51,5  +/-19,7 | N/A | 4/20 | N/A |
|  | B1 | 24 | 44,9  +/- 18,8 | N/A | 4/20 | N/A |
|  | B2 | 24 | 47,8  +/-17,5 | N/A | 2/22 | N/A |
| Lau et al.  1998 | A | 12 | "the two groups of patients were similar in ages, gender, height" | | | N/A |
|  | B | 10 |  |  |  | N/A |
| Siu et al.  2002 | A | 58 | 56,1 | N/A | 13/45 | N/A |
|  | B | 63 | 53,8 | N/A | 10/53 | N/A |
| Bertleff et al.  2009 | A | 49 | N/A | 59 | N/A | 23 |
|  | B | 52 | N/A | 66 | N/A | 24 |
| Shah et al.  2015 | A | 25 | N/A | 51  (27-61) | 4/21 | N/A |
|  | B | 25 | N/A | 50  (25-60) | 5/20 | N/A |
| Zedan et al.  2015 | A | 25 | 42  +/-13,4 | N/A | 6/18 | N/A |
|  | B | 25 | 40  +/- 9,4 | N/A | 7/14 | N/A |
| Zhang, Wang  2015 | A | 47 | 50,02 | N | 6/41 | N/A |
|  | B | 45 | 48,22 | N | 5/40 | N/A |
| Ge et al.  2016 | A | 61 | 46,5  +/- 18 | N | 7/54 | N/A |
|  | B | 58 | 46,4  +/- 20,4 | N/A | 9/49 | N/A |
| Abdullah et al.  2018 | A | 37 | 49,92  +/-12,82 | N/A | 6/31 | N/A |
|  | B | 33 | 46,97  +/- 14,78 | N/A | 5/28 | N/A |
| Srivastava et al.  2018 | A | 38 | N/A | N/A | 8/30 | N/A |
|  | B | 31 | N/A | N/A | 24/7 | N/A |
| Arroyo Vázquez et al.  2020 | C | 13 | N/A | 80  (38-87) | 7 / 6 | 24 (19-30) |
|  | A/B | 15 | N/A | 75  (23-91) | 8 / 7 | 28 (21-30) |
| Negm et al.  2022 | D | 50 | N/A | 49  (41-50) | 17/33 | N/A |
|  | A/B | 50 | N/A | 38  (27-54) | 14/36 | N/A |
|  | A | 10 | N/A | N/A | 4/6 | N/A |
|  | B | 40 | N/A | N/A | 10/20 | N/A |
| Khedr et al.  2023 | A | 74 | 47,56  +/- 11,99 | N/A | 12/62 | N/A |
|  | B | 66 | 44,88  +/- 15,44 | N/A | 10/56 | N/A |
| Saim et al.  2023 | A | N/A | N/A | N/A | N/A | N/A |
|  | B | N/A | N/A | N/A | N/A | N/A |
| Saleem et al.  2023 | A | 25 | 47,8  +/- 12, 79 | 47  (25:70) | 4/21 | N/A |
|  | B | 25 | 46,64+/- 12,47 | 53 (23:70) | 5/20 | N/A |

A: open surgical approach, A1: Open Suture, A2: Open Sutureless, B: Laparoscopic approach, B1: Laparoscopic Suture, B2: Laparoscopic Sutureless, A/B: surgical approach (open and laparoscopy), C: combined endoscopic and laparoscopic approach, D: combined radiologic and endoscopic approach, E: conservative approach, N/A: not available.

| Study | intervention/ treatment approach | Comorbidities (patients) | Alcohol history (yes/no) | Smoking history (yes/no) | Ulcer history (yes/no) | Use of Non-Steroidal Anti-Inflammatory Drugs (yes/no) interventions |
| --- | --- | --- | --- | --- | --- | --- |
| Crofts et al.  1989 | E | 19 | N/A | 33 | N/A | N/A |
|  | A | 14 | N/A | 32 | N/A | N/A |
| Lau et al.  1996 | A1 | N/A | N/A | N/A | N/A | N/A |
|  | A2 | N/A | N/A | N/A | N/A | N/A |
|  | B1 | N/A | N/A | N/A | N/A | N/A |
|  | B2 | N/A | N/A | N/A | N/A | N/A |
| Lau et al.  1998 | A | N/A | N/A | N/A | N/A | N/A |
|  | B | N/A | N/A | N/A | N/A | N/A |
| Siu et al.  2002 | A | N/A | 16 | 42 | 15 | 12 |
|  | B | N/A | 16 | 48 | 11 | 14 |
| Bertleff et al.  2009 | A | N/A | N/A | N/A | N/A | 13 |
|  | B | N/A | N/A | N/A | N/A | 17 |
| Shah et al.  2015 | A | N/A | N/A | N/A | 11 | N/A |
|  | B | N/A | N/A | N/A | 12 | N/A |
| Zedan et al.  2015 | A | N/A | N/A | N/A | 6/18 | 10/14 |
|  | B | N/A | N/A | N/A | 6/15 | 8/13 |
| Zhang, Wang  2015 | A | N/A | N/A | N/A | N/A | N/A |
|  | B | N/A | N/A | N/A | N/A | N/A |
| Ge et al.  2016 | A | 14 | 7/54 | 21/40 | 25 | N/A |
|  | B | 16 | 4/54 | 15/43 | 18 | N/A |
| Abdullah et al.  2018 | A | N/A | N/A | N/A | N/A | 8 |
|  | B | N/A | N/A | N/A | N/A | 8 |
| Srivastava et al.  2018 | A | N/A | N/A | N/A | N/A | N/A |
|  | B | N/A | N/A | N/A | N/A | N/A |
| Arroyo Vázquez et al.  2020 | C | N/A | N/A | N/A | N/A | N/A |
|  | A/B | N/A | N/A | N/A | N/A | N/A |
| Negm et al.  2022 | D | N/A | N/A | N/A | N/A | N/A |
|  | A/B | N/A | N/A | N/A | N/A | N/A |
|  | A | N/A | N/A | N/A | N/A | N/A |
|  | B | N/A | N/A | N/A | N/A | N/A |
| Khedr et al.  2023 | A | 30 | N/A | N/A | N/A | 8 |
|  | B | 18 | N/A | N/A | N/A | 8 |
| Saim et al.  2023 | A | N/A | N/A | N/A | N/A | N/A |
|  | B | N/A | N/A | N/A | N/A | N/A |
| Saleem et al.  2023 | A | N/A | 1 | 12 | 5 | 10 |
|  | B | N/A | 2 | 11 | 6 | 9 |

A: open surgical approach, A1: Open Suture, A2: Open Sutureless, B: Laparoscopic approach, B1: Laparoscopic Suture, B2: Laparoscopic Sutureless, A/B: surgical approach (open and laparoscopy), C: combined endoscopic and laparoscopic approach, D: combined radiologic and endoscopic approach, E: conservative approach, N/A: not available.

| Study | intervention/ treatment approach | median ASA-Score | ASA- Score 1  (patients) | ASA-Score 2 (patients) | ASA-Score 3 (patients) | ASA-Score 4 (patients) |
| --- | --- | --- | --- | --- | --- | --- |
| Crofts et al.  1989 | E | N/A | N/A | N/A | N/A | N/A |
|  | A | N/A | N/A | N/A | N/A | N/A |
| Lau et al.  1996 | A1 | N/A | N/A | N/A | N/A | N/A |
|  | A2 | N/A | N/A | N/A | N/A | N/A |
|  | B1 | N/A | N/A | N/A | N/A | N/A |
|  | B2 | N/A | N/A | N/A | N/A | N/A |
| Lau et al.  1998 | A | N/A | N/A | N/A | N/A | N/A |
|  | B | N/A | N/A | N/A | N/A | N/A |
| Siu et al.  2002 | A | N/A | 29 | 19 | 8 | 2 |
|  | B | N/A | 33 | 18 | 8 | 4 |
| Bertleff et al.  2009 | A | 1,5 | N/A | N/A | N/A | N/A |
|  | B | 1 | N/A | N/A | N/A | N/A |
| Shah et al.  2015 | A | N/A | N/A | N/A | N/A | N/A |
|  | B | N/A | N/A | N/A | N/A | N/A |
| Zedan et al.  2015 | A | N/A | N/A | N/A | N/A | N/A |
|  | B | N/A | N/A | N/A | N/A | N/A |
| Zhang, Wang  2015 | A | N/A | N/A | N/A | N/A | N/A |
|  | B | N/A | N/A | N/A | N/A | N/A |
| Ge et al.  2016 | A | N/A | 19 | 35 | 7 | 0 |
|  | B | N/A | 24 | 29 | 5 | 0 |
| Abdullah et al.  2018 | A | N/A | 31 | 8 | 7 | 6 |
|  | B | N/A | 16 | 1 | 1 | 0 |
| Srivastava et al.  2018 | A | N/A | N/A | N/A | N/A | N/A |
|  | B | N/A | N/A | N/A | N/A | N/A |
| Arroyo Vázquez et al.  2020 | C | N/A | 2 | 6 | 2 | 3 |
|  | A/B | N/A | 3 | 4 | 8 | 0 |
| Negm et al.  2022 | D | N/A | N/A | N/A | N/A | N/A |
|  | A/B | N/A | N/A | N/A | N/A | N/A |
|  | A | N/A | N/A | N/A | N/A | N/A |
|  | B | N/A | N/A | N/A | N/A | N/A |
| Khedr et al.  2023 | A | N/A | 44 | 12 | 8 | 10 |
|  | B | N/A | 50 | 6 | 8 | N/A |
| Saim et al.  2023 | A | N/A | N/A | N/A | N/A | N/A |
|  | B | N/A | N/A | N/A | N/A | N/A |
| Saleem et al.  2023 | A | N/A | 8 | 10 | 5 | 2 |
|  | B | N/A | 10 | 8 | 6 | 1 |

A: open surgical approach, A1: Open Suture, A2: Open Sutureless, B: Laparoscopic approach, B1: Laparoscopic Suture, B2: Laparoscopic Sutureless, A/B: surgical approach (open and laparoscopy), C: combined endoscopic and laparoscopic approach, D: combined radiologic and endoscopic approach, E: conservative approach, N/A: not available.

| Study | intervention/ treatment approach | APACHE II-Score (0-34 pts.) total | APACHE II-Score (0-34 pts) interventions | Previous surgeries in the upper abdominal area (patients) |
| --- | --- | --- | --- | --- |
| Crofts et al.  1989 | E | N/A | N/A | N/A |
|  | A | N/A | N/A | N/A |
| Lau et al.  1996 | A1 | N/A | 5 (3-17) | N/A |
|  | A2 | N/A | 6 (3-12) | N/A |
|  | B1 | N/A | 6 (0-12) | N/A |
|  | B2 | N/A | 6 (2-15) | N/A |
| Lau et al.  1998 | A | N/A | N/A | N/A |
|  | B | N/A | N/A | N/A |
| Siu et al.  2002 | A | N/A | N/A | N/A |
|  | B | N/A | N/A | N/A |
| Bertleff et al.  2009 | A | N/A | N/A | N/A |
|  | B | N/A | N/A | N/A |
| Shah et al.  2015 | A | N/A | N/A | N/A |
|  | B | N/A | N/A | N/A |
| Zedan et al.  2015 | A | N/A | N/A | N/A |
|  | B | N/A | N/A | N/A |
| Zhang, Wang  2015 | A | N/A | N/A | N/A |
|  | B | N/A | N/A | N/A |
| Ge et al.  2016 | A | N/A | N/A | 1 |
|  | B | N/A | N/A | 1 |
| Abdullah et al.  2018 | A | N/A | N/A | N/A |
|  | B | N/A | N/A | N/A |
| Srivastava et al.  2018 | A | N/A | N/A | N/A |
|  | B | N/A | N/A | N/A |
| Arroyo Vázquez et al.  2020 | C | N/A | N/A | N/A |
|  | A/B | N/A | N/A | N/A |
| Negm et al.  2022 | D | N/A | N/A | N/A |
|  | A/B | N/A | N/A | N/A |
|  | A | N/A | N/A | N/A |
|  | B | N/A | N/A | N/A |
| Khedr et al.  2023 | A | N/A | N/A | 6 |
|  | B | N/A | N/A | 12 |
| Saim et al.  2023 | A | N/A | N/A | N/A |
|  | B | N/A | N/A | N/A |
| Saleem et al.  2023 | A | N/A | N/A | N/A |
|  | B | N/A | N/A | N/A |

A: open surgical approach, A1: Open Suture, A2: Open Sutureless, B: Laparoscopic approach, B1: Laparoscopic Suture, B2: Laparoscopic Sutureless, A/B: surgical approach (open and laparoscopy), C: combined endoscopic and laparoscopic approach, D: combined radiologic and endoscopic approach, E: conservative approach, N/A: not available.

| Study | Inter-vention/ treatment approach | Symptom duration (h) <12h | Symptom duration (h) >12h | Symptom duration (h) <24h | Symptom duration (h) >24h | Average symptom duration (h) | Median symptom duration (h) (range) |
| --- | --- | --- | --- | --- | --- | --- | --- |
| Crofts et al.  1989 | E | N/A | N/A | N/A | N/A | 10,5 +/- 16 | N/A |
|  | A | N/A | N/A | N/A | N/A | 12,7 +/- 14 | N/A |
| Lau et al.  1996 | A1 | N/A | N/A | N/A | N/A | N/A | N/A |
|  | A2 | N/A | N/A | N/A | N/A | N/A | N/A |
|  | B1 | N/A | N/A | N/A | N/A | N/A | N/A |
|  | B2 | N/A | N/A | N/A | N/A | N/A | N/A |
| Lau et al.  1998 | A | N/A | N/A | N/A | N/A | 10 | N/A |
|  | B | N/A | N/A | N/A | N/A | 13,5 | N/A |
| Siu et al.  2002 | A | N/A | N/A | N/A | 6 | N/A | N/A |
|  | B | N/A | N/A | N/A | 1 | N/A | N/A |
| Bertleff et al.  2009 | A | N/A | N/A | N/A | N/A | 11 | N/A |
|  | B | N/A | N/A | N/A | N/A | 11 | N/A |
| Shah et al.  2015 | A | N/A | N/A | N/A | N/A | N/A | N/A |
|  | B | N/A | N/A | N/A | N/A | N/A | N/A |
| Zedan et al.  2015 | A | N/A | N/A | N/A | 3 | 14,7 +/-10 | N/A |
|  | B | N/A | N/A | N/A | 3 | 14 +/- 8,4 | N/A |
| Zhang, Wang  2015 | A | N/A | N/A | N/A | N/A | N/A | N/A |
|  | B | N/A | N/A | N/A | N/A | N/A | N/A |
| Ge et al.  2016 | A | N/A | N/A | N/A | N/A | N/A | 9 (7-15) |
|  | B | N/A | N/A | N/A | N/A | N/A | 8 (6-11) |
| Abdullah et al.  2018 | A | N/A | N/A | N/A | N/A | 30,68 +/- 8,85 | N/A |
|  | B | N/A | N/A | N/A | N/A | 29,45 +/- 9,79 | N/A |
| Srivastava et al.  2018 | A | N/A | N/A | N/A | N/A | N/A | N/A |
|  | B | N/A | N/A | N/A | N/A | N/A | N/A |
| Arroyo Vázquez  et al.  2020 | C | 13 | 7 | 0 | 0 | N/A | N/A |
|  | A/B | 15 | 5 | 0 | 0 | N/A | N/A |
| Negm et al.  2022 | D | N/A | N/A | N/A | N/A | N/A | overall: 12-36h |
|  | A/B | N/A | N/A | N/A | N/A | N/A | N/A |
|  | A | N/A | N/A | N/A | N/A | N/A | N/A |
|  | B | N/A | N/A | N/A | N/A | N/A | N/A |
| Khedr et al.  2023 | A | N/A | N/A | N/A | N/A | 29,77 +/- 6,90 | N/A |
|  | B | N/A | N/A | N/A | N/A | 27,56 +/- 8,67 | N/A |
| Saim et al.  2023 | A | N/A | N/A | N/A | N/A | N/A | N/A |
|  | B | N/A | N/A | N/A | N/A | N/A | N/A |
| Saleem et al.  2023 | A | N/A | N/A | N/A | 11 | N/A | N/A |
|  | B | N/A | N/A | N/A | 10 | N/A | N/A |

A: open surgical approach, A1: Open Suture, A2: Open Sutureless, B: Laparoscopic approach, B1: Laparoscopic Suture, B2: Laparoscopic Sutureless, A/B: surgical approach (open and laparoscopy), C: combined endoscopic and laparoscopic approach, D: combined radiologic and endoscopic approach, E: conservative approach, N/A: not available.

*Intraoperative findings of the included studies*

| Study | intervention/ treatment approach | Median size of perforation (mm) <10 | Median size of perforation (mm) >10 | Median size of perforation (mm) |
| --- | --- | --- | --- | --- |
| Crofts et al.  1989 | E | N/A | N/A | N/A |
|  | A | N/A | N/A | N/A |
| Lau et al.  1996 | A1 | N/A | N/A | 5 (2-25) |
|  | A2 | N/A | N/A | 5 (2-10) |
|  | B1 | N/A | N/A | 6 (1-20) |
|  | B2 | N/A | N/A | 5 (2-15) |
| Lau et al.  1998 | A | N/A | N/A | N/A |
|  | B | N/A | N/A | N/A |
| Siu et al.  2002 | A | N/A | N/A | N/A |
|  | B | N/A | N/A | N/A |
| Bertleff et al.  2009 | A | N/A | N/A | 7 |
|  | B | N/A | N/A | 10 |
| Shah et al.  2015 | A | N/A | N/A | N/A |
|  | B | N/A | N/A | N/A |
| Zedan et al.  2015 | A | N/A | N/A | N/A |
|  | B | N/A | N/A | N/A |
| Zhang, Wang  2015 | A | N/A | N/A | N/A |
|  | B | N/A | N/A | N/A |
| Ge et al.  2016 | A | N/A | N/A | 4 (3-6) |
|  | B | N/A | N/A | 5 (5-8) |
| Abdullah et al.  2018 | A | N/A | N/A | N/A |
|  | B | N/A | N/A | N/A |
| Srivastava et al.  2018 | A | N/A | N/A | N/A |
|  | B | N/A | N/A | N/A |
| Arroyo Vázquez et al.  2020 | C | N/A | N/A | N/A |
|  | A/B | N/A | N/A | N/A |
| Negm et al.  2022 | D | N/A | N/A | N/A |
|  | A/B | N/A | N/A | N/A |
|  | A | N/A | N/A | N/A |
|  | B | N/A | N/A | N/A |
| Khedr et al.  2023 | A | N/A | N/A | N/A |
|  | B | N/A | N/A | N/A |
| Saim et al.  2023 | A | N/A | N/A | N/A |
|  | B | N/A | N/A | N/A |
| Saleem et al.  2023 | A | N/A | N/A | 8 (4:16) |
|  | B | N/A | N/A | 9(4:15) |

A: open surgical approach, A1: Open Suture, A2: Open Sutureless, B: Laparoscopic approach, B1: Laparoscopic Suture, B2: Laparoscopic Sutureless, A/B: surgical approach (open and laparoscopy), C: combined endoscopic and laparoscopic approach, D: combined radiologic and endoscopic approach, E: conservative approach, N/A: not available.

| Study | intervention/ treatment approach | Average size of perforation (mm) +/- SD | Size of perforation <1 (cm) | Size of perforation >1 (cm) |
| --- | --- | --- | --- | --- |
| Crofts et al.  1989 | E | N/A | N/A | N/A |
|  | A | N/A | N/A | N/A |
| Lau et al.  1996 | A1 | N/A | N/A | N/A |
|  | A2 | N/A | N/A | N/A |
|  | B1 | N/A | N/A | N/A |
|  | B2 | N/A | N/A | N/A |
| Lau et al.  1998 | A | N/A | N/A | N/A |
|  | B | N/A | N/A | N/A |
| Siu et al.  2002 | A | 4,7 (3,0) | N/A | N/A |
|  | B | 5,2 (4,9) | N/A | N/A |
| Bertleff et al.  2009 | A | N/A | N/A | N/A |
|  | B | N/A | N/A | N/A |
| Shah et al.  2015 | A | N/A | 21 | 4 |
|  | B | N/A | 20 | 5 |
| Zedan et al.  2015 | A | 5,5 +/- 2,4 | N/A | N/A |
|  | B | 5 +/- 1,5 | N/A | N/A |
| Zhang, Wang  2015 | A | 6,95 +/- 3,11 | N/A | N/A |
|  | B | 6,82 +/- 3,08 | N/A | N/A |
| Ge et al.  2016 | A | N/A | N/A | N/A |
|  | B | N/A | N/A | N/A |
| Abdullah et al.  2018 | A | 5,68 +/- 2,1 | N/A | N/A |
|  | B | 5,67 +/- 1,67 | N/A | N/A |
| Srivastava et al.  2018 | A | N/A | N/A | N/A |
|  | B | N/A | N/A | N/A |
| Arroyo Vázquez et al.  2020 | C | N/A | N/A | N/A |
|  | A/B | N/A | N/A | N/A |
| Negm et al.  2022 | D | N/A | N/A | N/A |
|  | A/B | N/A | N/A | N/A |
|  | A | N/A | N/A | N/A |
|  | B | N/A | N/A | N/A |
| Khedr et al.  2023 | A | 4,99 +/-2,12 | N/A | N/A |
|  | B | 5,22 +/- 2,12 | N/A | N/A |
| Saim et al.  2023 | A | N/A | N/A | N/A |
|  | B | N/A | N/A | N/A |
| Saleem et al.  2023 | A | 8,96+/-4,16 | N/A | N/A |
|  | B | 8,8+/-2,61 | N/A | N/A |

A: open surgical approach, A1: Open Suture, A2: Open Sutureless, B: Laparoscopic approach, B1: Laparoscopic Suture, B2: Laparoscopic Sutureless, A/B: surgical approach (open and laparoscopy), C: combined endoscopic and laparoscopic approach, D: combined radiologic and endoscopic approach, E: conservative approach, N/A: not available.

| Study | intervention/ treatment approach | Location of perforation (stomach unspecified) | Location of the perforation (stomach (pyloric-area)) | Location of the perforation (pre-pyloric) | Location of the perforation (duodenal) |
| --- | --- | --- | --- | --- | --- |
| Crofts et al.  1989 | E | 1 | N/A | N/A | 7 |
|  | A | 2 | N/A | N/A | 40 |
| Lau et al.  1996 | A1 | 3 | 2 | N/A | 16 |
|  | A2 | 1 | 2 | N/A | 21 |
|  | B1 | 1 | 4 | N/A | 20 |
|  | B2 | 1 | 3 | N/A | 19 |
| Lau et al.  1998 | A | N/A | N/A | N/A | N/A |
|  | B | N/A | N/A | N/A | N/A |
| Siu et al.  2002 | A | 0 | 4 | 6 | 48 |
|  | B | 1 | 2 | 15 | 45 |
| Bertleff et al.  2009 | A | N/A | 12 | 22 | 14 |
|  | B | N/A | 8 | 19 | 20 |
| Shah et al.  2015 | A | N/A | 2 | 10 | 13 |
|  | B | N/A | 3 | 5 | 16 |
| Zedan et al.  2015 | A | N/A | N/A | N/A | N/A |
|  | B | N/A | N/A | N/A | N/A |
| Zhang, Wang  2015 | A | 15 | N/A | N/A | 32 |
|  | B | 11 | N/A | N/A | 34 |
| Ge et al.  2016 | A | 30 | N/A | N/A | 31 |
|  | B | 19 | N/A | N/A | 39 |
| Abdullah et al.  2018 | A | 8 | N/A | 8 | N/A |
|  | B | 0 | N/A | 7 | N/A |
| Srivastava et al.  2018 | A | N/A | N/A | N/A | N/A |
|  | B | N/A | N/A | N/A | N/A |
| Arroyo Vázquez et al.  2020 | C | N/A | N/A | N/A | 13 |
|  | A/B | N/A | N/A | N/A | 15 |
| Negm et al.  2022 | D | 21 | N/A | N/A | 29 |
|  | A/B | 15 | N/A | N/A | 35 |
|  | A | N/A | N/A | N/A | N/A |
|  | B | N/A | N/A | N/A | N/A |
| Khedr et al.  2023 | A | 16 | N/A | 16 | 42 |
|  | B | 0 | N/A | 14 | 52 |
| Saim et al.  2023 | A | N/A | N/A | N/A | N/A |
|  | B | N/A | N/A | N/A | N/A |
| Saleem et al.  2023 | A | 3 | 2 | N/A | 9 |
|  | B | 4 | 2 | N/A | 7 |

A: open surgical approach, A1: Open Suture, A2: Open Sutureless, B: Laparoscopic approach, B1: Laparoscopic Suture, B2: Laparoscopic Sutureless, A/B: surgical approach (open and laparoscopy), C: combined endoscopic and laparoscopic approach, D: combined radiologic and endoscopic approach, E: conservative approach, N/A: not available

| Study | intervention/treatment approach | Median bloodloss (ml) | Average bloodloss (ml) +/- SD | Success of closure of the finding (yes/no) | Konversion to A (yes/no) |
| --- | --- | --- | --- | --- | --- |
| Crofts et al.  1989 | E | N/A | N/A | N/A | 3 |
|  | A | N/A | N/A | N/A | - |
| Lau et al.  1996 | A1 | N/A | N/A | N/A | - |
|  | A2 | N/A | N/A | N/A | - |
|  | B1 | N/A | N/A | N/A | 7 |
|  | B2 | N/A | N/A | N/A | 4 |
| Lau et al.  1998 | A | N/A | N/A | N/A | - |
|  | B | N/A | N/A | N/A | 3 |
| Siu et al.  2002 | A | N/A | N/A | N/A | - |
|  | B | N/A | N/A | N/A | 9 |
| Bertleff et al.  2009 | A | 10 | N/A | N/A | - |
|  | B | 10 | N/A | N/A | 4 |
| Shah et al.  2015 | A | N/A | N/A | N/A | - |
|  | B | N/A | N/A | N/A | 0 |
| Zedan et al.  2015 | A | N/A | N/A | N/A | - |
|  | B | N/A | N/A | N/A | 4 |
| Zhang, Wang  2015 | A | N/A | 95,23 +/- 14,79 | N/A | N/A |
|  | B | N/A | 15,76 +/- 2,38 | N/A | N/A |
| Ge et al.  2016 | A | N/A | N/A | N/A | - |
|  | B | N/A | N/A | N/A | 1 |
| Abdullah et al.  2018 | A | N/A | N/A | N/A | - |
|  | B | N/A | N/A | N/A | 15 |
| Srivastava et al.  2018 | A | N/A | N/A | N/A | N/A |
|  | B | N/A | N/A | N/A | N/A |
| Arroyo Vázquez et al.  2020 | C | N/A | N/A | N/A | 0 |
|  | A/B | N/A | N/A | N/A | 10 |
| Negm et al.  2022 | D | N/A | N/A | 48 | N/A |
|  | A/B | N/A | N/A | 45 | N/A |
|  | A | N/A | N/A | N/A | N/A |
|  | B | N/A | N/A | N/A | N/A |
| Khedr et al.  2023 | A | N/A | N/A | N/A | 0 |
|  | B | N/A | N/A | N/A | 15 |
| Saim et al.  2023 | A | N/A | 124,2 | N/A | N/A |
|  | B | N/A | 43 | N/A | N/A |
| Saleem et al.  2023 | A | N/A | N/A | N/A | 0 |
|  | B | N/A | N/A | N/A | 2 |

A: open surgical approach, A1: Open Suture, A2: Open Sutureless, B: Laparoscopic approach, B1: Laparoscopic Suture, B2: Laparoscopic Sutureless, A/B: surgical approach (open and laparoscopy), C: combined endoscopic and laparoscopic approach, D: combined radiologic and endoscopic approach, E: conservative approach, N/A: not available.

*Descriptive outcomes of the studies*

1. *Morbidity (Clavien-Dindo-Classification)*

| Study | intervention/ treatment approach | Morbidity Clavien-Dindo grade 0-1 (patients) | Morbidity Clavien-Dindo grade 2 (patients) | Morbidity Clavien-Dindo grade 3a & b (patients) | Morbidity Clavien-Dindo grade 4a & b (patients) | Morbidity Clavien-Dindo grade 5 (patients) |
| --- | --- | --- | --- | --- | --- | --- |
| Arroyo Vázquez et al.  2020 | C | 6 | 0 | 3 | 3 | 5 |
|  | A/B | 9 | 3 | 1 | 2 | 0 |
| Saleem et al.  2023 | A | 11 | 9 | 3 | 2 | N/A |
|  | B | 15 | 7 | 2 | 1 | N/A |

A: open surgical approach B: Laparoscopic approach, A/B: surgical approach (open and laparoscopy), C: combined endoscopic and laparoscopic approach, N/A: not available.

1. *Postoperative complications: total p.o. complications, fever, respiratory insufficiency*

| Study | intervention/ treatment approach | Total p.o. complications | Total p.o. complications (patients) | p.o. complications (Fever) | p.o. complications (respiratory insufficiency) |
| --- | --- | --- | --- | --- | --- |
| Crofts et al.  1989 | E | 20 | N/A | N/A | N/A |
|  | A | 17 | N/A | N/A | N/A |
| Lau et al.  1996 | A1 | 5 | N/A | N/A | N/A |
|  | A2 | 5 | N/A | N/A | N/A |
|  | B1 | 5 | N/A | N/A | N/A |
|  | B2 | 6 | N/A | N/A | N/A |
| Bertleff et al.  2009 | A | 24 | 15 | N/A | 3 |
|  | B | 12 | 9 | N/A | 1 |
| Zhang, Wang  2015 | A | 12 | N/A | N/A | N/A |
|  | B | 2 | N/A | N/A | N/A |
| Ge et al.  2016 | A | 9 | 7 | 0 | 1 |
|  | B | 8 | 6 | 0 | 1 |
| Srivastava et al.  2018 | A | N/A | 15 | N/A | N/A |
|  | B | N/A | 3 | N/A | N/A |
| Arroyo Vázquez et al.  2020 | C | N/A | N/A | 0 | N/A |
|  | A/B | N/A | N/A | 2 | N/A |
| Negm et al.  2022 | D | 12 | N/A | 10 | N/A |
|  | A/B | 29 | N/A | 11 | N/A |
|  | A | N/A | N/A | N/A | N/A |
|  | B | N/A | N/A | N/A | N/A |
| Khedr et al.  2023 | A | 10 | 38 | N/A | N/A |
|  | B | 10 | 6 | N/A | N/A |
| Saim et al.  2023 | A | N/A | N/A | N/A | N/A |
|  | B | N/A | N/A | N/A | N/A |
| Saleem et al.  2023 | A | 18 | 14 | N/A | N/A |
|  | B | 19 | 9 | N/A | N/A |

A: open surgical approach, A1: Open Suture, A2: Open Sutureless, B: Laparoscopic approach, B1: Laparoscopic Suture, B2: Laparoscopic Sutureless, A/B: surgical approach (open and laparoscopy), C: combined endoscopic and laparoscopic approach, D: combined radiologic and endoscopic approach, E: conservative approach, N/A: not available, p.o.: postoperative.

1. *Postoperative complications: ARDS, cardiac problems, sepsis, fascia dehiscence*

| Study | intervention/ treatment approach | ARDS | Cardiac problems | Sepsis | Fascia dehiscence |
| --- | --- | --- | --- | --- | --- |
| Lau et al.  1996 | A1 | N/A | 0 | N/A | N/A |
|  | A2 | N/A | 1 | N/A | N/A |
|  | B1 | N/A | 0 | N/A | N/A |
|  | B2 | N/A | 1 | N/A | N/A |
| Siu et al.  2002 | A | 1 | N/A | N/A | N/A |
|  | B | 0 | N/A | N/A | N/A |
| Bertleff et al.  2009 | A | 1 | 2 | 1 | 1 |
|  | B | 0 | 2 | 3 | 0 |
| Zedan et al.  2015 | A | N/A | N/A | 3 | N/A |
|  | B | N/A | N/A | 2 | N/A |
| Ge et al.  2016 | A | N/A | N/A | 1 | 1 |
|  | B | N/A | N/A | 1 | 0 |
| Abdullah et al.  2018 | A | N/A | N/A | N/A | 9 |
|  | B | N/A | N/A | N/A | 0 |

ARDS: Acute Respiratory Distress Syndrome, A: open surgical approach, A1: Open Suture, A2: Open Sutureless, B: Laparoscopic approach, B1: Laparoscopic Suture, B2: Laparoscopic Sutureless, N/A: not available.

1. *Postoperative complications: urinary tract infections, incisional hernia, cerebrovascular events*

| Study | intervention/ treatment approach | Urinary tract infection | Incisional hernia | Cerebrovascular events |
| --- | --- | --- | --- | --- |
| Crofts et al.  1989 | E | N/A | N/A | 1 |
|  | A | N/A | N/A | 1 |
| Lau et al.  1996 | A1 | 1 | 1 | N/A |
|  | A2 | 0 | 0 | N/A |
|  | B1 | 0 | 0 | N/A |
|  | B2 | 0 | 0 | N/A |
| Bertleff et al.  2009 | A | 2 | 1 | 1 |
|  | B | 0 | 0 | 0 |
| Zhang, Wang  2015 | A | N/A | N/A | 1 |
|  | B | N/A | N/A | 0 |
| Ge et al.  2016 | A | N/A | 1 | N/A |
|  | B | N/A | 0 | N/A |
| Negm et al.  2022 | D | N/A | 0 | N/A |
|  | A/B | N/A | 3 | N/A |
|  | A | N/A | 3 | N/A |
|  | B | N/A | 0 | N/A |

A: open surgical approach, A1: Open Suture, A2: Open Sutureless, B: Laparoscopic approach, B1: Laparoscopic Suture, B2: Laparoscopic Sutureless, A/B: surgical approach (open and laparoscopy), D: combined radiologic and endoscopic approach, E: conservative approach, N/A: not available.

1. *Postoperative complications: dysphagia, abdominal collection*

| Study | intervention/ treatment approach | p.o. complications (dysphagia) | p.o. complications (abdominal collection) |
| --- | --- | --- | --- |
| Siu et al.  2002 | A | N/A | 0 |
|  | B | N/A | 2 |
| Abdullah et al.  2018 | A | N/A | 3 |
|  | B | N/A | 1 |
| Negm et al.  2022 | A | N/A | 2 |
|  | B | N/A | 1 |
| Saleem et al.  2023 | A | N/A | 1 |
|  | B | N/A | 2 |

A: open surgical approach, B: Laparoscopic approach, N/A: not available

1. *Duration of surgery, duration of nasogastric tube placement, time to resume to diet*

| Study | intervention/ treatment approach | Average duration of surgery (minutes) +/- SD | Median duration of surgery (minutes) | Median duration of nasogastric tube placement (days) (range) | Median time to resume to diet (days) (range) | Average time to resume to diet +/- SD (days) |
| --- | --- | --- | --- | --- | --- | --- |
| Lau et al.  1996 | A1 | N/A | 56,9 +/-47,6 | 2 (1-13) | 4 (3-16) | N/A |
|  | A2 | N/A | 50,8 +/- 38,6 | 3 (1-17) | 4 (3-19) | N/A |
|  | B1 | N/A | 112,9 +/- 44,1 | 2 (1-4) | 4 (3-7) | N/A |
|  | B2 | N/A | 74,8 +/- 24,3 | 3 (2-10) | 4 (2-11) | N/A |
| Lau et al.  1998 | A | N/A | 35 | N/A | "not significantly different in either group" | N/A |
|  | B | N/A | 96 | N/A | "not significantly different in either group" | N/A |
| Siu et al.  2002 | A | 52,3 (24,8) | N/A | 3 (1-8) | 5 (3-24) | N/A |
|  | B | 42 (25,1) | N/A | 3 (2-33) | 4 (3-35) | N/A |
| Bertleff et al.  2009 | A | N/A | 50 | 3 | N/A | N/A |
|  | B | N/A | 75 | 2 | N/A | N/A |
| Shah et al.  2015 | A | 90 | N/A | N/A | N/A | N/A |
|  | B | 60 | N/A | N/A | N/A | N/A |
| Zedan et al.  2015 | A | 110 +/-13 | N/A | N/A | N/A | 6,2 +/- 2 |
|  | B | 145 +/- 19 | N/A | N/A | N/A | 4,5 +/- 1,4 |
| Zhang, Wang  2015 | A | 91,06 | N/A | N/A | N/A | N/A |
|  | B | 118,21 | N/A | N/A | N/A | N/A |
| Ge et al.  2016 | A | N/A | 75 (60-90) | 4 (3-4) | 4 (4-5) | N/A |
|  | B | N/A | 70 (60-90) | 3,5 (3-4) | 4 (4-5) | N/A |
| Abdullah et al.  2018 | A | 46,54 +/- 15,92 | N/A | N/A | N/A | N/A |
|  | B | 61,67 +/- 18,55 | N/A | N/A | N/A | N/A |
| Srivastava et al.  2018 | A | 60,32+/-7,56 | N/A | N/A | N/A | N/A |
|  | B | 101,90+/-12,84 | N/A | N/A | N/A | N/A |
| Arroyo Vázquez et al.  2020 | C | 68 | N/A | N/A | 21 | N/A |
|  | A/B | 92 | N/A | N/A | N/A | N/A |
| Negm et al.  2022 | D | 17,5 | N/A | N/A | N/A | N/A |
|  | A/B | 50 | N/A | N/A | N/A | N/A |
|  | A | 30-50 | N/A | N/A | N/A | N/A |
|  | B | 17-40 | N/A | N/A | N/A | N/A |
| Khedr et al.  2023 | A | 47,23 +/-1,77 | N/A | N/A | N/A | N/A |
|  | B | 59,98 +/- 19,23 | N/A | N/A | N/A | N/A |
| Saim et al.  2023 | A | 67,3 | N/A | N/A | N/A | N/A |
|  | B | 116,2 | N/A | N/A | N/A | N/A |
| Saleem et al.  2023 | A | 100  +/-16,45 | 100 (75:125) | N/A | 5 (3:6) | 4,83  +/-0,87 |
|  | B | 117,4  +/-13,0 | 115 (90:150) | N/A | 2 (2:5) | 2,84  +/-1,14 |

A: open surgical approach, A1: Open Suture, A2: Open Sutureless, B: Laparoscopic approach, B1: Laparoscopic Suture, B2: Laparoscopic Sutureless, A/B: surgical approach (open and laparoscopy), D: combined radiologic and endoscopic approach, N/A: not available.

*7. Postoperative pain*

| Study | intervention/ treatment approach | Average postoperative hospital stay (days) total | Average postoperative pain (as defined in the respective studies) +/- SD | Average postoperative pain (as defined in the respective studies) +/- SD Day 1 | Average postoperative pain (as defined in the respective studies) +/- SD Day 2 |
| --- | --- | --- | --- | --- | --- |
| Siu et al.  2002 | A | N/A | N/A | 6,4  (1,3) | 3,3  (1,1) |
|  | B | N/A | N/A | 3,5  (1,4) | 1,6  (1,1) |
| Zedan et al.  2015 | A | 8,9  +/- 3,3 | N/A | 7  +/- 0,9 | N/A |
|  | B | 6,9  +/- 2,2 | N/A | 4,4  +/-0,8 | N/A |
| Zhang, Wang  2015 | A | 5,15  +/- 1,52 | 6,69  +/- 1,21 | N/A | N/A |
|  | B | 9.09  +/- 2,21 | 3,01  +/- 1,06 | N/A | N/A |
| Srivastava et al.  2018 | A | 12,08  +/-4,82 | N/A | N/A | N/A |
|  | B | 8,42  +/-1,44 | N/A | N/A | N/A |
| Khedr et al.  2023 | A | 9,65  +/- 3,12 | N/A | N/A | N/A |
|  | B | 6,11  +/- 2,02 | N/A | N/A | N/A |
| Saim et al.  2023 | A | 15 | N/A | N/A | N/A |
|  | B | 4,3 | N/A | N/A | N/A |
| Saleem et al.  2023 | A | 9,8  +/-2,33 | 73,24  +/-12,95 | N/A | N/A |
|  | B | 6,0  8+/-2,86 | 46,72  +/-13,22 | N/A | N/A |

A: open surgical approach, B: Laparoscopic approach, N/A: not available.

1. *Postoperative pain*

| Study | intervention/ treatment approach | median postoperative pain (as defined in the respective studies) +/- SD Day 1 | Median postoperative pain (as defined in the respective studies) Day 3 | Median postoperative pain (as defined in the respective studies) Day 7 | Median postoperative pain (as defined in the respective studies) Day 28 |
| --- | --- | --- | --- | --- | --- |
| Lau et al.  1996 | A1 | 5 (2-9) | N/A | N/A | N/A |
|  | A2 | 5 (2-9) | 4 (2-11) | N/A | N/A |
|  | B1 | 4 (2-8) | N/A | N/A | N/A |
|  | B2 | 4 (1-9) | N/A | N/A | N/A |
| Bertleff et al.  2009 | A | 5,15 | 3 | 1,85 | 0 |
|  | B | 3,8 | 2,1 | 1 | 0,3 |

A: open surgical approach, A1: Open Suture, A2: Open Sutureless, B: Laparoscopic approach, B1: Laparoscopic Suture, B2: Laparoscopic Sutureless, N/A: not available

1. *Reoperation/Reintervention, perioperative pain medication requirement, p.o. opioid usage*

| Study | intervention/ treatment approach | Reoperation/ Reintervention (yes/no) | Perioperative pain medication requirement (days) | Average p.o. opioid usage (days) +/- SD | Median p.o. opioid usage (doses) (range) |
| --- | --- | --- | --- | --- | --- |
| Ge et al.  2016 | A | 0 | N/A | N/A | N/A |
|  | B | 1 | N/A | N/A | N/A |
| Abdullah et al.  2018 | A | 3 | N/A | N/A | N/A |
|  | B | 1 | N/A | N/A | N/A |
| Khedr et al.  2023 | A | 6 | N/A | N/A | N/A |
|  | B | 2 | N/A | N/A | N/A |
| Saim et al.  2023 | A | N/A | 2,8 | N/A | N/A |
|  | B | N/A | 4,9 | N/A | N/A |
| Saleem et al.  2023 | A | 2 | N/A | N/A | N/A |
|  | B | 1 | N/A | N/A | N/A |

A: open surgical approach, B: Laparoscopic approach, N/A: not available, p.o.: postoperative

1. *Cosmetic outcome, total cost, return to normal physical activity, median intravenous infusion administration*

| Study | intervention/ treatment approach | Cosmetic outcome  (VAS score for scar appearance) | Total cost (EUR) | Return to normal physical activity (days) +/- SD | Median intravenous infusion administration (days) (range) |
| --- | --- | --- | --- | --- | --- |
| Lau et al.  1996 | A1 | N/A | N/A |  | 4 (2-19) |
|  | A2 | N/A | N/A |  | 4 (2-17) |
|  | B1 | N/A | N/A |  | 4 (2-8) |
|  | B2 | N/A | N/A |  | 4 (2-11) |
| Siu et al.  2002 | A | N/A | N/A | 26,1 (15,1) | 4 (2-35) |
|  | B | N/A | N/A | 10,4 (6,9) | 4 (2-26) |
| Bertleff et al.  2009 | A | 7,7 | N/A | N/A | N/A |
|  | B | 5,5 | N/A | N/A | N/A |
| Shah et al.  2015 | A | N/A | N/A | 10 | N/A |
|  | B | N/A | N/A | 5 | N/A |
| Zedan et al.  2015 | A | "good: 2; accepted: 6; not accepted: 16" | N/A | 20,5 +/- 3,9 | N/A |
|  | B | "good: 15; accepted: 4; not accepted: "2" | N/A | 14 +/- 1,9 | N/A |
| Zhang, Wang  2015 | A | N/A | 2493,28 | N/A | N/A |
|  | B | N/A | 3123,18 | N/A | N/A |
| Ge et al.  2016 | A | N/A | 1786,08 | N/A | N/A |
|  | B | N/A | 1931,11 | N/A | N/A |
| Khedr et al.  2023 | A | “Due to the increased rates of SSI, wound dehiscence, and lengthy laparotomy incisions with consequent unsightly scars in the open approach of our experience, there was a significant difference in the cosmetic outcome between the laparoscopic and open approaches.” | N/A | N/A | N/A |
|  | B |  | N/A | N/A | N/A |
| Saim et al.  2023 | A | N/A | N/A | 32,07 | N/A |
|  | B | N/A | N/A | 13,33 | N/A |
| Saleem et al.  2023 | A | "good: 13; accepted: 6; not accepted: 4" | N/A | 24,78+/-6,60 | 25 (15:40) |
|  | B | "good: 20; accepted: 4; not accepted: 1“ | N/A | 15,6+/-2,60 | 15 (12:22) |

A: open surgical approach, A1: Open Suture, A2: Open Sutureless, B: Laparoscopic approach, B1: Laparoscopic Suture, B2: Laparoscopic Sutureless, N/A: not available.

1. *Decrease in leucocyte count*

| Study | intervention/ treatment approach | Decrease in leukocyte count (preoperative) (x10^9/l) | Decrease in leukocyte count (Day 1 p.o.) (x10^9/l) | Decrease in leukocyte count (Day 2 p.o.) (x10^9/l) | Decrease in leukocyte count (Day 3 p.o.) (x10^9/l) | Decrease in leukocyte count (Day 4 p.o.) (x10^9/l) |
| --- | --- | --- | --- | --- | --- | --- |
| Ge et al.  2016 | A | 11,8 +/- 4,0 | N/A | N/A | N/A | N/A |
|  | B | 11,9 +/-4,2 | N/A | N/A | N/A | N/A |
| Arroyo Vázquez et al.  2020 | C | 15 | 13 | 10 | 8 | 8 |
|  | A/B | 17 | 13 | 10 | 9 | 8 |

A: open surgical approach, B: Laparoscopic approach, A/B: surgical approach (open and laparoscopy), C: combined laparoscopic and endoscopic approach, N/A: not available.

1. *Decrease in leucocyte count*

| Study | intervention/ treatment approach | Decrease in CRP (preoperative) (ng/ml) | Decrease in CRP (Day 1 p.o.) (ng/ml) | Decrease in CRP (Day 2 p.o.) (ng/ml) | Decrease in CRP (Day 3 p.o.) (ng/ml) | Decrease in CRP (Day 4 p.o.) (ng/ml) |
| --- | --- | --- | --- | --- | --- | --- |
| Lau et al.  1998 | A | 2 ug/dl | 17 ug/dl | 12 ug/dl | 9 ug/dl | N/A |
|  | B | 4 ug/dl | 16 ug/dl | 18 ug/dl | 13 ug/dl | N/A |
| Arroyo Vázquez et al.  2020 | C | 80 | 270 | 310 | 260 | 130 |
|  | A/B | 10 | 220 | 250 | 150 | 100 |

A: open surgical approach, B: Laparoscopic approach, A/B: surgical approach (open and laparoscopy), C: combined laparoscopic and endoscopic approach, N/A: not available.

**Supplemental material 2: The study features, patient characteristics, follow- up and outcomes**
