# Supplementary material for: Approaches for the treatment of perforated peptic ulcers: a network meta-analysis of randomized controlled trials
Source: Langenbecks Arch Surg. 2025 Sep 5;410(1):266. doi: 10.1007/s00423-025-03848-9 (PMC12413338; doi:10.1007/s00423-025-03848-9)
Supplement: Supplementary file 3 — (228KB) [file 423_2025_3848_MOESM3_ESM.docx]

1. Mortality

**Table** Heterogeneity mortality

| Heterogeneity | Value |
| --- | --- |
| tau^2^ | 0 |
| tau | 0 |
| I^2^ | 0% [0.0%; 62.4%] |

**Table** test of heterogeneity mortality

| Tests of heterogeneity | Q | d.f. | p-value |
| --- | --- | --- | --- |
| Total | 7.03 | 9 | 0.6342 |
| Within designs | 6.79 | 8 | 0.5592 |
| Between designs | 0.24 | 1 | 0.6275 |

**Forest Plot** Comparison A versus B for mortality


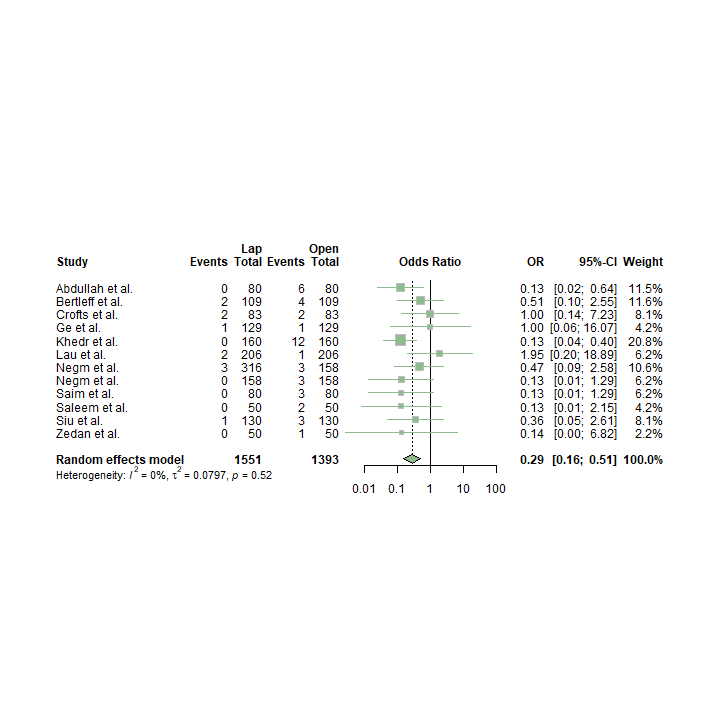


A, Open surgical approach; B, Laparoscopic approach; CI, Confidence interval; OR, Odds ratio

**Funnel Plot** Mortality


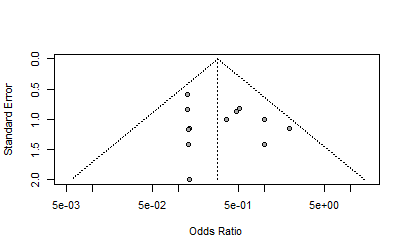


**Summary of Findings Table** Mortality

| comparison | OR | lower | upper | CIR | CIR lower | CIR upper |
| --- | --- | --- | --- | --- | --- | --- |
| A | 1.000 | 1.000 | 1.000 | 33 | 33 | 33 |
| B | 0.360 | 0.172 | 0.751 | 13 | 6 | 25 |
| C | 0.117 | 0.004 | 3.212 | 4 | 1 | 99 |
| D | 0.119 | 0.007 | 2.164 | 5 | 1 | 69 |
| E | 1.000 | 0.138 | 7.272 | 33 | 5 | 199 |

The mean incidence of death in the study arms A is 0.033 (3.3%). From 1000 patients treated with A 33 patients will reach the event. The SoF Table Mortality shows the relative effects (OR+ 95% CI) and the corresponding intervention risk (CIR) of the the respective intervention compared to A and an assumed comparator risk (ACR) of 3.3%. The calcuations are conducted for the estimated OR as well as for the CIs by the formulas provided in the Cochrane Handbook chapter 14.1.51 (1)

A, Open surgical approach; B, Laparoscopic approach; C, Combined laparoscopic – endoscopic approach; CIR, Corresponding intervention risk; D, Combined endoscopic-radiologic approach; E, Conservative approach; OR, Odds ratio

1. Patients With Complications

**Forest Plot** Comparison A versus B for patients with complications


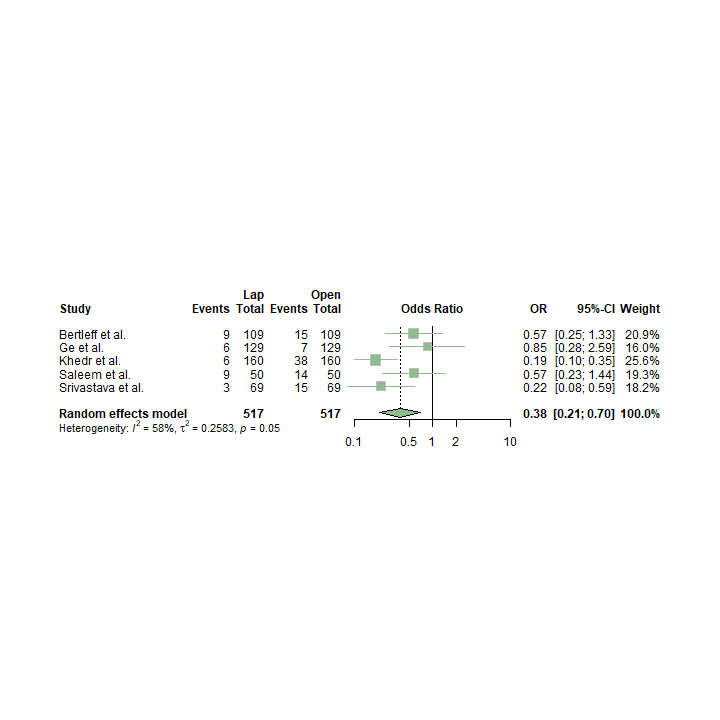


A, Open surgical approach; B, Laparoscopic approach; CI, Confidence interval; OR, Odds ratio

**Funnel Plot** Patients with complications


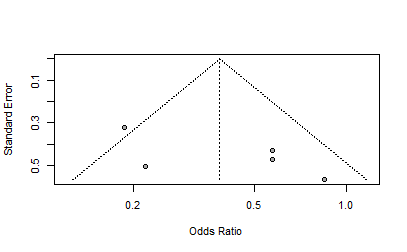


1. Leakage

**Table** NMA for leakage

| Treatment | OR | 95%-CI | p-value |
| --- | --- | --- | --- |
| A | - | - | - |
| B | 0.7544 | [0.2424; 2.3483] | 0.6266 |
| C | 0.7544 | [0.0134; 42.3390] | 0.8909 |
| D | 0.5222 | [0.0305; 8.9505] | 0.6540 |

A, Open surgical approach; B, Laparoscopic approach; C, Combined laparoscopic – endoscopic approach; CI, Confidence interval; D, Combined endoscopic-radiologic approach; NMA, Network meta-analysis; OR, Odds ratio

**Table** Heterogeneity leakage

| Heterogeneity | Value |
| --- | --- |
| tau^2^ | 1.8394 |
| tau | 1.3562 |
| I^2^ | 60.8% [21.7%; 80.4%] |

**Table** test of heterogeneity leakage

| Tests of heterogeneity | Q | d.f. | p-value |
| --- | --- | --- | --- |
| Total | 22.96 | 9 | 0.0063 |
| Within designs | 20.66 | 8 | 0.0081 |
| Between designs | 2.30 | 1 | 0.1292 |

**Netgraph** for leakage


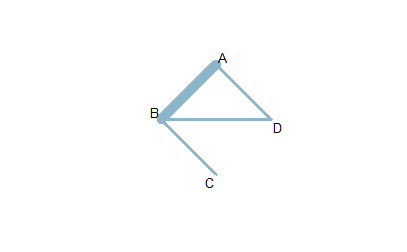


A, Open surgical approach; B, Laparoscopic approach; C, Combined laparoscopic – endoscopic approach; D, Combined endoscopic-radiologic approach

**Forest Plot** NMA for leakage


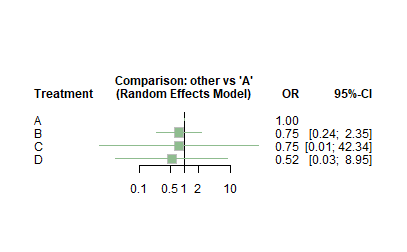


A, Open surgical approach; B, Laparoscopic approach; C, Combined laparoscopic – endoscopic approach; CI, Confidence interval; D, Combined endoscopic-radiologic approach; NMA, Network meta-analysis; OR, Odds ratio

**Forest Plot** Comparison A versus B for leakage


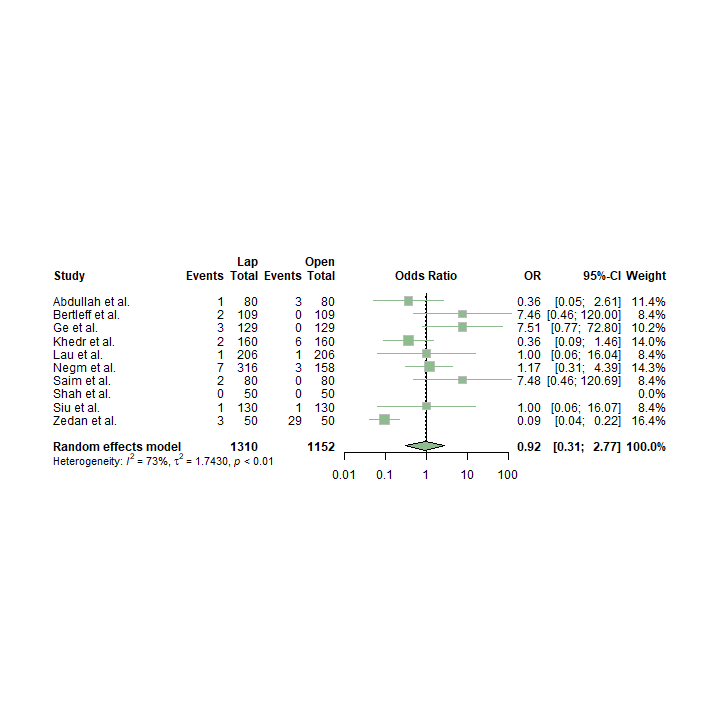


A, Open surgical approach; B, Laparoscopic approach; CI, Confidence interval; OR, Odds ratio

**Funnel Plot** Leakage


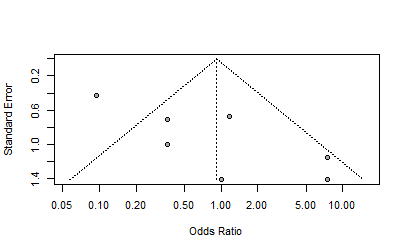


**Summary Of Findings Table** leakage

| comparison | OR | lower | upper | CIR | CIR lower | CIR upper |
| --- | --- | --- | --- | --- | --- | --- |
| A | 1.000 | 1.000 | 1.000 | 69 | 69 | 69 |
| B | 0.754 | 0.242 | 2.348 | 53 | 18 | 149 |
| C | 0.754 | 0.013 | 42.339 | 53 | 1 | 759 |
| D | 0.522 | 0.030 | 8.950 | 38 | 3 | 399 |

A, Open surgical approach; B, Laparoscopic approach; C, Combined laparoscopic – endoscopic approach; CIR, Corresponding intervention risk; D, Combined endoscopic-radiologic approach; OR, Odds ratio

1. Pneumonia

**Table** NMA for pneumonia

| Treatment | OR | 95%-CI | p-value |
| --- | --- | --- | --- |
| A | - | - | - |
| B | 0.5761 | [0.2462; 1.3480] | 0.2035 |
| C | 0.1876 | [0.0061; 5.7855] | 0.3388 |
| D | 0.0944 | [0.0048; 1.8413] | 0.1194 |

A, open surgical approach; B, laparoscopic approach; C, combined laparoscopic – endoscopic approach; CI, confidence interval; D, combined endoscopic-radiologic approach; OR, odds ratio; NMA, network meta-analysis

**Table** heterogeneity pneumonia

| Heterogeneity | Value |
| --- | --- |
| tau^2^ | 0.1591 |
| tau | 0.3988 |
| I^2^ | 13.6% [0.0%; 78.1%] |

**Table** test of heterogeneity pneumonia

| Tests of heterogeneity | Q | d.f. | p-value |
| --- | --- | --- | --- |
| Total | 5.79 | 5 | 0.3274 |
| Within designs | 4.54 | 4 | 0.3380 |
| Between designs | 1.25 | 1 | 0.2636 |

**Netgraph** Pneumonia


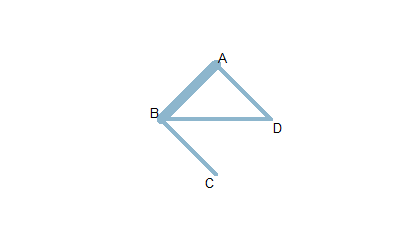


A, Open surgical approach; B, Laparoscopic approach; C, Combined laparoscopic – endoscopic approach; D, Combined endoscopic-radiologic approach

**Forest Plot** NMA for pneumonia


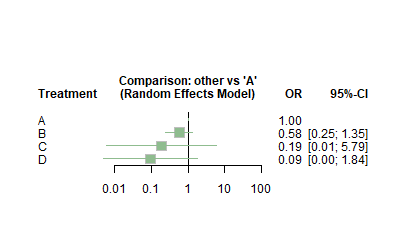


A, Open surgical approach; B, Laparoscopic approach; C, Combined laparoscopic – endoscopic approach; CI, Confidence interval; D, Combined endoscopic-radiologic approach; OR, Odds ratio; NMA, Network meta-analysis

**Forest Plot** Comparison A versus B for pneumonia


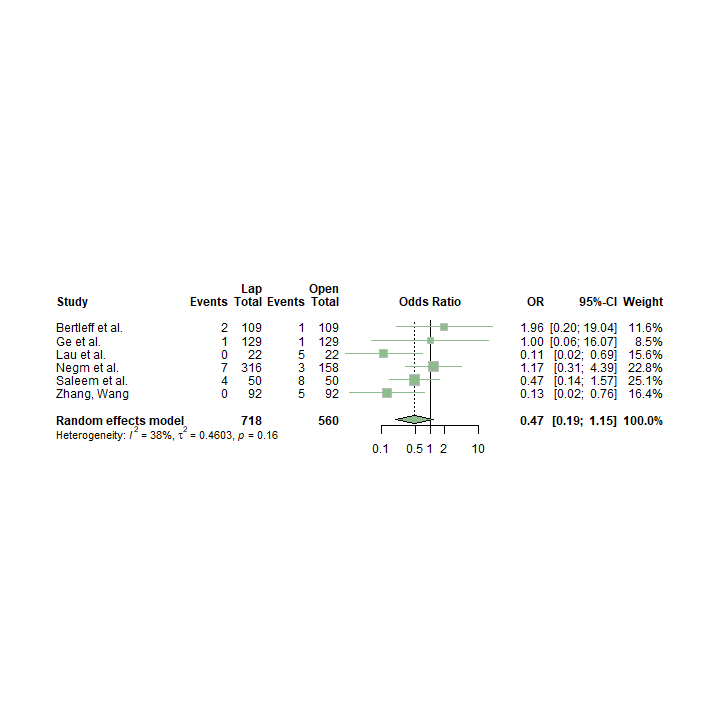


A, Open surgical approach; B, Laparoscopic approach; CI, Confidence interval; OR, Odds ratio

**Funnel Plot** Pneumonia


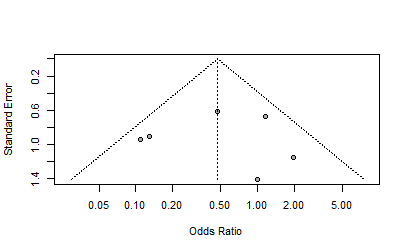


**Summary Of Findings Table** for pneumonia

| comparison | OR | lower | upper | CIR | CIR lower | CIR upper |
| --- | --- | --- | --- | --- | --- | --- |
| A | 1.000 | 1.000 | 1.000 | 80 | 80 | 80 |
| B | 0.576 | 0.246 | 1.348 | 48 | 21 | 105 |
| C | 0.188 | 0.006 | 5.786 | 17 | 1 | 335 |
| D | 0.094 | 0.005 | 1.841 | 9 | 1 | 138 |

A, Open surgical approach; B, Laparoscopic approach; C, Combined laparoscopic – endoscopic approach; CIR, Corresponding intervention risk; D, Combined endoscopic-radiologic approach; OR, Odds ratio

1. Abscess

**Table** NMA for abscess

| Treatment | OR | 95%-CI | p-value |
| --- | --- | --- | --- |
| A | - | - | - |
| B | 0.6277 | [0.1732; 2.2745] | 0.4784 |
| C | 1.2861 | [0.0703; 23.5390] | 0.8653 |
| D | 0.0880 | [0.0030; 2.5492] | 0.1570 |
| E | 3.1558 | [0.4854; 20.5177] | 0.2289 |

A, Open surgical approach; B, Laparoscopic approach; C, Combined laparoscopic – endoscopic approach; CI, Confidence interval; D, Combined endoscopic-radiologic approach; E, Conservative approach; NMA, Network meta-analysis; OR, Odds ratio

**Table** heterogeneity abscess

| Heterogeneity | Value |
| --- | --- |
| tau^2^ | 0.2203 |
| tau | 0.4693 |
| I^2^ | 12.3% [0.0%; 86.6%] |

**Table** test of heterogeneity abscess

| Tests of heterogeneity | Q | d.f. | p-value |
| --- | --- | --- | --- |
| Total | 3.42 | 3 | 0.3311 |
| Within designs | 3.42 | 3 | 0.3311 |
| Between designs | 0.00 | 0 | -- |

**Netgraph** Abscess


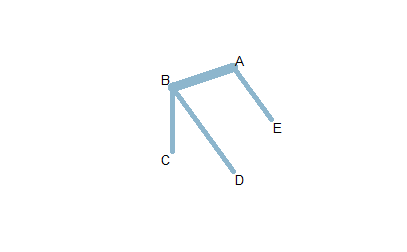


A, Open surgical approach; B, Laparoscopic approach; C, Combined laparoscopic – endoscopic approach; D, Combined endoscopic-radiologic approach; E, Conservative approach

**Forest Plot** NMA for abscess


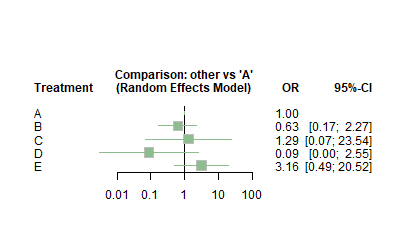


A, Open surgical approach; B, Laparoscopic approach; C, Combined laparoscopic – endoscopic approach; CI, Confidence interval; D, Combined endoscopic-radiologic approach; E, Conservative approach; NMA, Network meta-analysis; OR, Odds ratio

**Forest Plot** Comparison A versus B for abscess


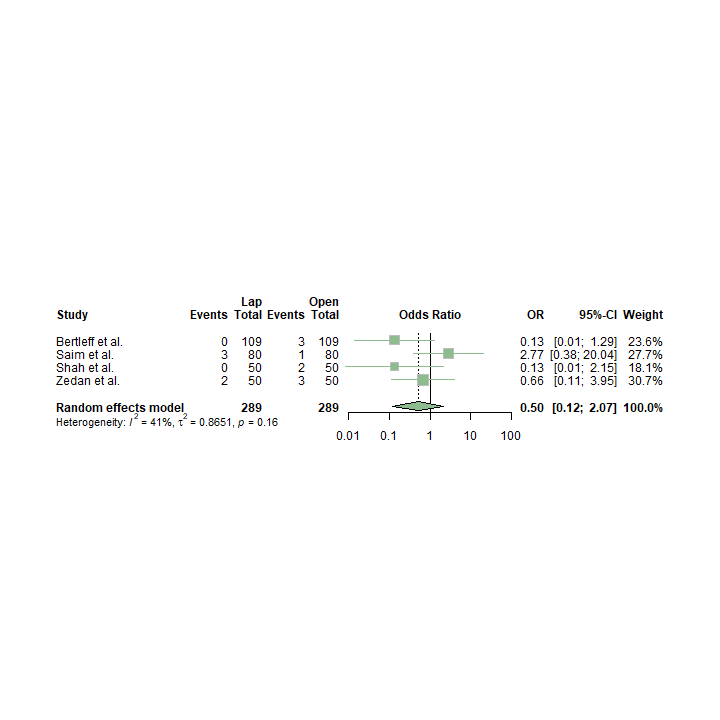


A, Open surgical approach; B, Laparoscopic approach; CI, Confidence interval; OR, Odds ratio

**Funnel Plot** Abscess


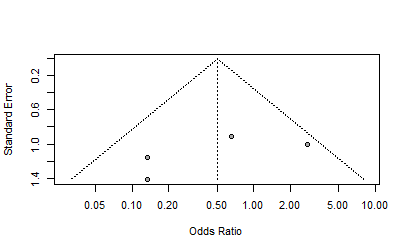


**Summary of Findings Table** Abscess

| comparison | OR | lower | upper | CIR | CIR lower | CIR upper |
| --- | --- | --- | --- | --- | --- | --- |
| A | 1.000 | 1.000 | 1.000 | 33 | 33 | 33 |
| B | 0.628 | 0.173 | 2.274 | 21 | 6 | 73 |
| C | 1.286 | 0.070 | 23.539 | 43 | 3 | 446 |
| D | 0.088 | 0.003 | 2.549 | 3 | 1 | 81 |
| E | 3.156 | 0.485 | 20.518 | 98 | 17 | 412 |

A, Open surgical approach; B, Laparoscopic approach; C, Combined laparoscopic – endoscopic approach; CIR, Corresponding intervention risk; D, Combined endoscopic-radiologic approach; E, Conservative approach; OR, Odds ratio

1. Ileus

**Forest Plot** Comparison A versus B For Ileus


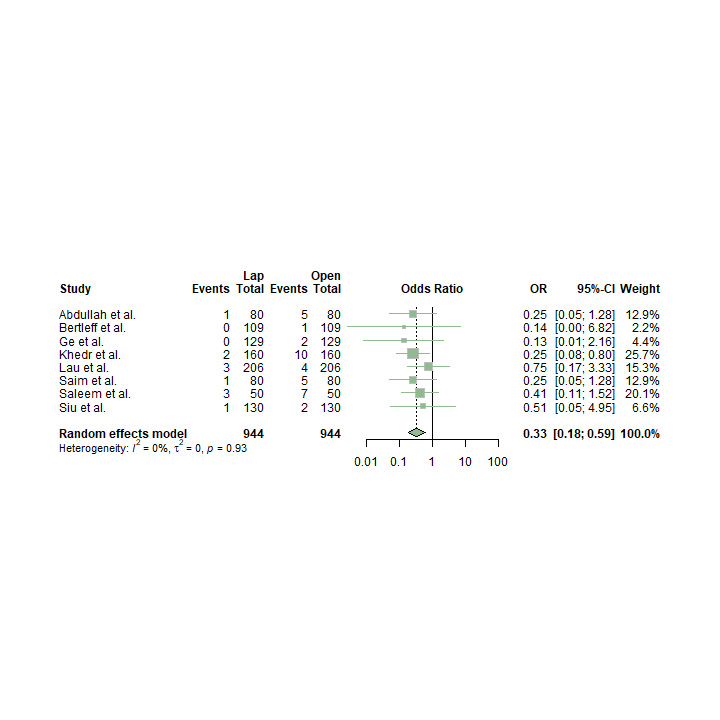


A, Open surgical approach; B, Laparoscopic approach; CI, Confidence interval; OR, Odds ratio

**Funnel Plot** Ileus


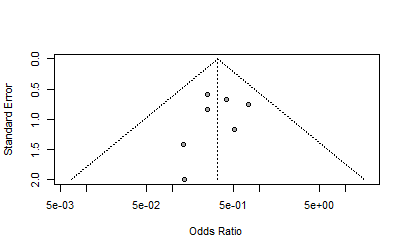


**Summary Of Findings Table** Ileus

| comparison | OR | lower | upper | CIR | CIR lower | CIR upper |
| --- | --- | --- | --- | --- | --- | --- |
| A | 1.000 | 1.000 | 1.000 | 48 | 48 | 48 |
| B | 0.328 | 0.166 | 0.648 | 17 | 9 | 32 |

A, Open surgical approach; B, Laparoscopic approach; CIR, Corresponding intervention risk; OR, Odds ratio

1. Wound Infection

**Table** heterogeneity wound infection

| Heterogeneity | Value |
| --- | --- |
| Tau^2^ | 0 |
| tau | 0 |
| I^2^ | 0% [0.0%; 58.3%] |

**Table** tests of heterogeneity wound infection

| Tests of heterogeneity | Q | d.f. | p-value |
| --- | --- | --- | --- |
| Total | 9.73 | 11 | 0.5550 |
| Within designs | 9.73 | 11 | 0.5550 |
| Between designs | 0.00 | 0 | -- |

**Netgraph** Wound Infection


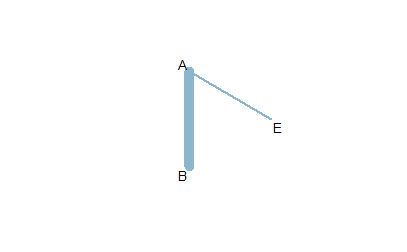


A, Open surgical approach; B, Laparoscopic approach; E, Conservative approach

**Forest Plot** Comparison A versus B for wound infection


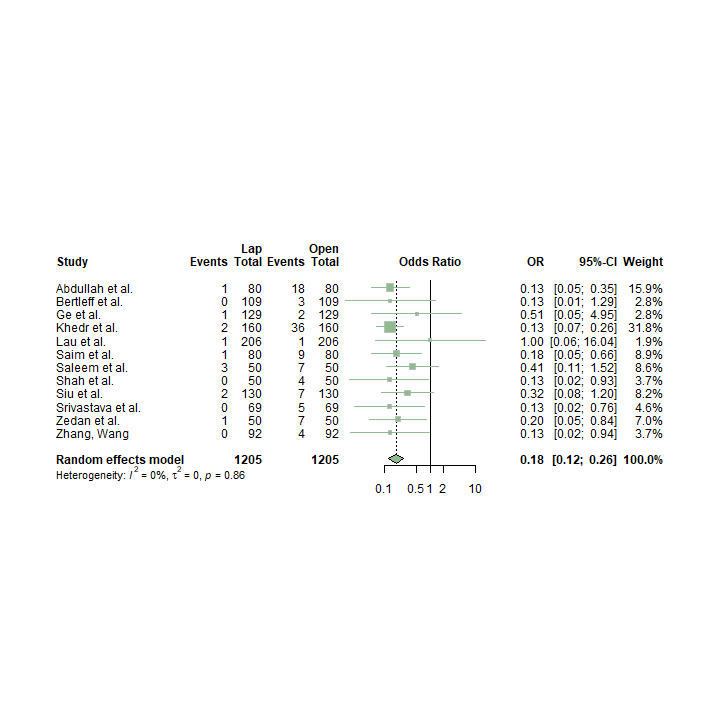


A, Open surgical approach; B, Laparoscopic approach; CI, Confidence interval; OR, Odds ratio

**Funnel Plot** wound infection


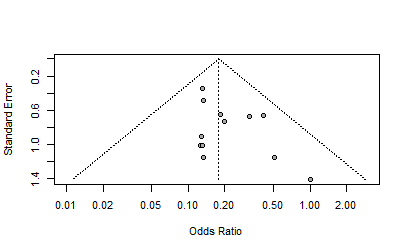


**Summary Of Findings Table** wound infection

| comparison | OR | lower | upper | CIR | CIR lower | CIR upper |
| --- | --- | --- | --- | --- | --- | --- |
| A | 1.000 | 1.000 | 1.000 | 90 | 90 | 90 |
| B | 0.149 | 0.082 | 0.271 | 15 | 9 | 27 |
| E | 1.000 | 0.138 | 7.272 | 90 | 14 | 419 |

A, Open surgical approach; B, Laparoscopic approach; E, Conservative approach; CIR, Corresponding intervention risk; OR, Odds ratio

1. Hernia

**Table** NMA for Hernia

| Treatment | OR | 95%-CI | p-value |
| --- | --- | --- | --- |
| A | - | - | - |
| B | 0.4229 | [0.1322; 1.3531] | 0.1470 |
| D | 0.1294 | [0.0069; 2.4317] | 0.1718 |

A, Open surgical approach; B, Laparoscopic approach; CI, Confidence interval; D, Combined endoscopic-radiologic approach; NMA, Network meta-analysis; OR, Odds ratio

**Table** heterogeneity hernia

| Heterogeneity | Value |
| --- | --- |
| tau^2^ | 0 |
| tau | 0 |
| I^2^ | 0% [0.0%; 84.7%] |

**Table** tests of heterogeneity hernia

| Tests of heterogeneity | Q | d.f. | p-value |
| --- | --- | --- | --- |
| Total | 0.11 | 3 | 0.9905 |
| Within designs | 0.00 | 2 | 1.0000 |
| Between designs | 0.11 | 1 | 0.7389 |

**Netgraph** Hernia


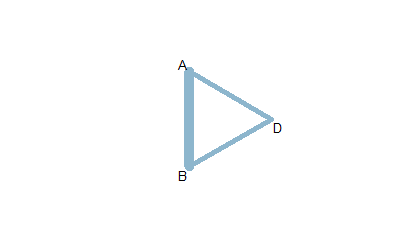


A, Open surgical approach; B, Laparoscopic approach; D, Combined endoscopic-radiologic approach

**Forest Plot** NMA for Hernia


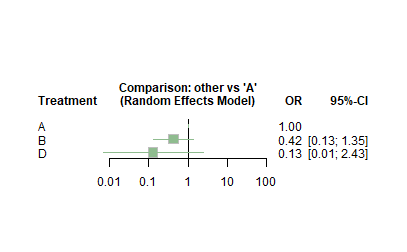


A, Open surgical approach; B, Laparoscopic approach; CI, Confidence interval; D, Combined endoscopic-radiologic approach; OR, Odds ratio; NMA, Network meta-analysis

**Forest Plot** Comparison A versus B for hernia


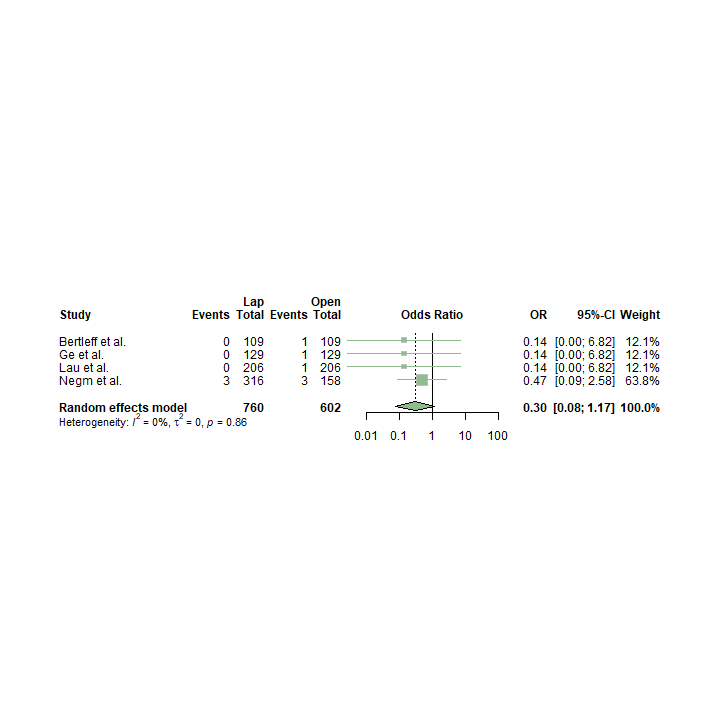


A, Open surgical approach; B, Laparoscopic approach; CI, Confidence interval; OR, Odds ratio

**Funnel Plot** hernia


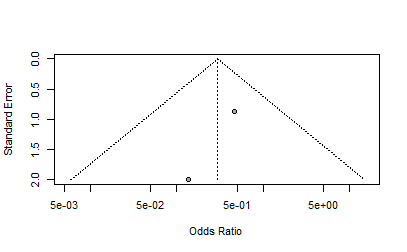


**Summary Of Findings Table** hernia

| comparison | OR | lower | upper | CIR | CIR lower | CIR lower |
| --- | --- | --- | --- | --- | --- | --- |
| A | 1.000 | 1.000 | 1.000 | 10 | 10 | 10 |
| B | 0.423 | 0.132 | 1.353 | 5 | 2 | 14 |
| D | 0.129 | 0.007 | 2.432 | 2 | 1 | 24 |

A, Open surgical approach; B, Laparoscopic approach; CIR, Corresponding intervention risk; D, Combined endoscopic-radiologic approach; E, Conservative approach; OR, Odds ratio

1. Organ Failure

**Table** NMA for organ failure

| Treatment | OR | 95%-CI | p-value |
| --- | --- | --- | --- |
| A | - | - | - |
| B | 0.5581 | [0.1669; 1.8658] | 0.3436 |
| C | 0.8581 | [0.0949; 7.7573] | 0.8916 |
| D | 0.2092 | [0.0107; 4.0985] | 0.3027 |
| E | 9.4528 | [0.5008; 178.4224] | 0.1340 |

A, Open surgical approach; B, Laparoscopic approach; C, Combined laparoscopic – endoscopic approach; CI, Confidence interval; D, Combined endoscopic-radiologic approach; E, Conservative approach; NMA, Network meta-analysis; OR, Odds ratio

**Table** heterogeneity organ failure

| Heterogeneity | Value |
| --- | --- |
| tau^2^ | 0 |
| tau | 0 |
| I^2^ | 0% [0.0%; 84.7%] |

**Table** tests of heterogeneity organ failure

| Tests of heterogeneity | Q | d.f. | p-value |
| --- | --- | --- | --- |
| Total | 0.82 | 3 | 0.8447 |
| Within designs | 0.79 | 2 | 0.6739 |
| Between designs | 0.03 | 1 | 0.8612 |

**Netgraph** organ failure


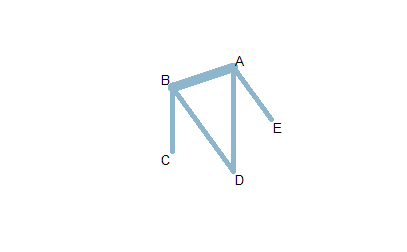


A, Open surgical approach; B, Laparoscopic approach; C, Combined laparoscopic – endoscopic approach; D, Combined endoscopic-radiologic approach; E, Conservative approach

**Forest Plot** NMA organ failure


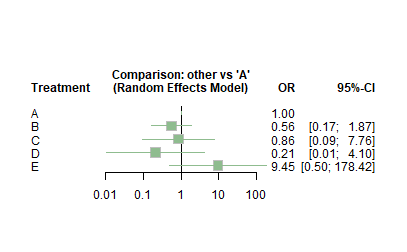


A, Open surgical approach; B, Laparoscopic approach; C, Combined laparoscopic – endoscopic approach; CI, Confidence interval; D, Combined endoscopic-radiologic approach; E, Conservative approach; OR, Odds ratio; NMA, Network meta-analysis

**Forest Plot** Comparison A versus B for organ failure


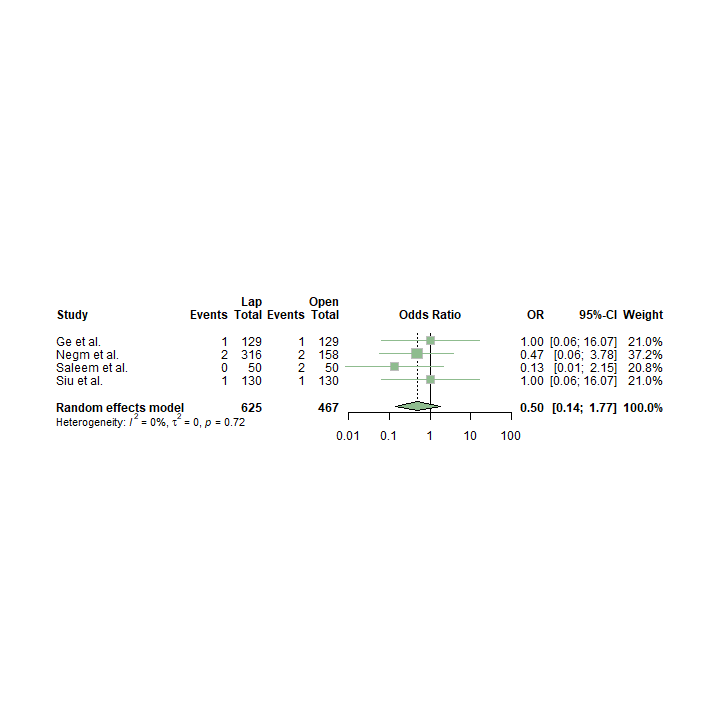


A, Open surgical approach; B, Laparoscopic approach; CI, Confidence interval; OR, Odds ratio

**Funnel Plot** organ failure


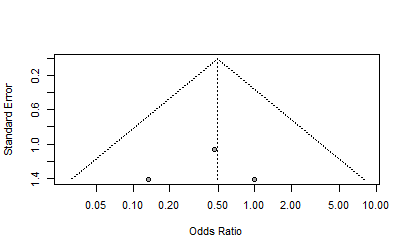


**Summary Of Findings Table** for organ failure

| comparison | OR | lower | upper | CIR | CIR lower | CIR upper |
| --- | --- | --- | --- | --- | --- | --- |
| A | 1.000 | 1.000 | 1.000 | 14 | 14 | 14 |
| B | 0.558 | 0.167 | 1.866 | 8 | 3 | 26 |
| C | 0.858 | 0.095 | 7.757 | 13 | 2 | 100 |
| D | 0.209 | 0.011 | 4.098 | 3 | 1 | 55 |
| E | 9.453 | 0.501 | 178.422 | 119 | 8 | 717 |

A, Open surgical approach; B, Laparoscopic approach; C, Combined laparoscopic – endoscopic approach; CIR, Corresponding intervention risk; D, Combined endoscopic-radiologic approach; E, Conservative approach; OR, Odds ratio

1. Chest Complications

**Table** NMA chest complications

| Treatment | OR | 95%-CI | p-value |
| --- | --- | --- | --- |
| A | - | - | - |
| B | 0.3010 | [0.0677; 1.3389] | 0.1149 |
| E | 0.5450 | [0.0359; 8.2656] | 0.6617 |

A, Open surgical approach; B, Laparoscopic approach; CI, Confidence interval; E, Conservative approach; NMA, Network meta-analysis; OR, Odds ratio

**Table** heterogeneity chest complications

| Heterogeneity | Value |
| --- | --- |
| tau^2^ | 1.6713 |
| tau | 1.2928 |
| I^2^ | 60.7% [0.0%; 85.3%] |

**Table** tests of heterogeneity chest complications

| Tests of heterogeneity | Q | d.f. | p-value |
| --- | --- | --- | --- |
| Total | 10.18 | 4 | 0.0375 |
| Within designs | 10.18 | 4 | 0.0375 |
| Between designs | 0.00 | 0 | -- |

**Netgraph** chest complications


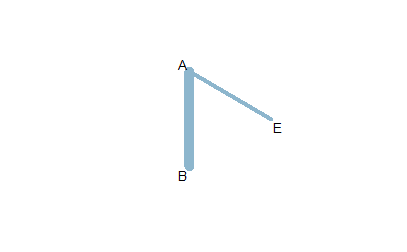


A, Open surgical approach; B, Laparoscopic approach; E, Conservative approach

**Forest Plot** NMA chest complications


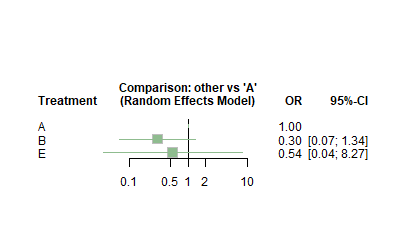


A, Open surgical approach; B, Laparoscopic approach; CI, Confidence interval; E, conservative approach; NMA, Network meta-analysis; OR, Odds ratio

**Forest Plot** Comparison A versus B chest complications


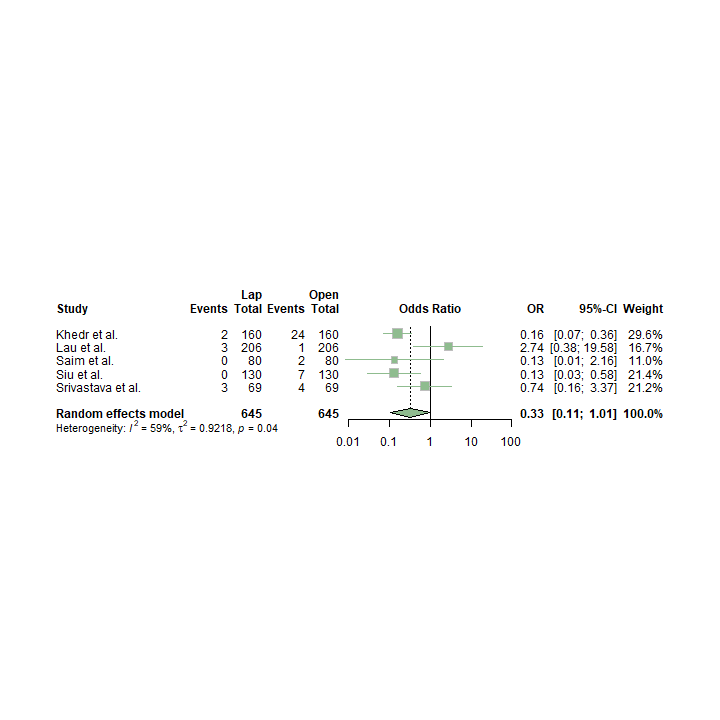


A, Open surgical approach; B, Laparoscopic approach; CI, Confidence interval; OR, Odds ratio

**Funnel Plot** Chest Complications


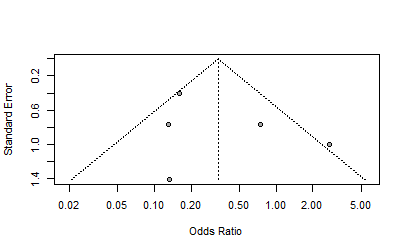


**Summary Of Findings Table** chest complications

| comparison | OR | lower | upper | CIR | CIR lower | CIR upper |
| --- | --- | --- | --- | --- | --- | --- |
| A | 1.000 | 1.000 | 1.000 | 73 | 73 | 73 |
| B | 0.301 | 0.068 | 1.339 | 24 | 6 | 96 |
| E | 0.545 | 0.036 | 8.266 | 42 | 3 | 395 |

A, Open surgical approach; B, Laparoscopic approach; CIR, Corresponding intervention risk; E, Conservative approach; OR, Odds ratio

1. Hospital Stay

**Table** NMA hospital stay

| Treatment | MD | 95%-CI | p-value |
| --- | --- | --- | --- |
| A | . | . | . |
| B | -0.1988 | [-2.7696; 2.3720] | 0.8795 |
| C | 0.8012 | [-7.2461; 8.8484] | 0.8453 |
| E | 4.2000 | [-2.1234; 10.5234] | 0.1930 |

A, Open surgical approach; B, Laparoscopic approach; C, Combined laparoscopic – endoscopic approach; CI, Confidence interval; E, Conservative approach; NMA, Network meta-analysis; OR, Odds ratio

**Table** heterogeneity hospital stay

| Heterogeneity | Value |
| --- | --- |
| Tau^2^ | 8.0642 |
| tau | 2.8398 |
| I^2^ | 97.8% [96.6%; 98.6%] |

**Table** tests of heterogeneity hospital stay

| Tests of heterogeneity | Q | d.f. | p-value |
| --- | --- | --- | --- |
| Total | 183.44 | 4 | < 0.0001 |
| Within designs | 183.44 | 4 | < 0.0001 |
| Between designs | 0.00 | 0 | -- |

**Netgraph** hospital stay


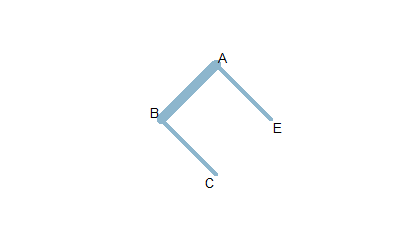


A, Open surgical approach; B, Laparoscopic approach; C, Combined laparoscopic – endoscopic approach; E, Conservative approach

**Forest Plot** NMA hospital stay


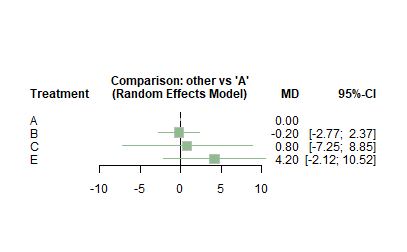


A, Open surgical approach; B, Laparoscopic approach; C, Combined laparoscopic – endoscopic approach; CI, Confidence interval; E, Conservative approach; MD, Mean difference; NMA, Network meta-analysis

**Supplemental Material 3:** Results of the network meta-analysis, pairwise comparisons, heterogeneity, netgraphs, funnel plots, Summary of Findings Tables

References

1. Higgins JPT, Thomas J, Chandler J, Cumpston M, Li T, Page MJ, Welch VA (editors). Cochrane Handbook for Systematic Reviews of Interventions. Cochrane; 2023.
