# Supplementary figures and images for: Approaches for the treatment of perforated peptic ulcers: a network meta-analysis of randomized controlled trials
Source: Langenbecks Arch Surg. 2025 Sep 5;410(1):266. doi: 10.1007/s00423-025-03848-9 (PMC12413338; doi:10.1007/s00423-025-03848-9)

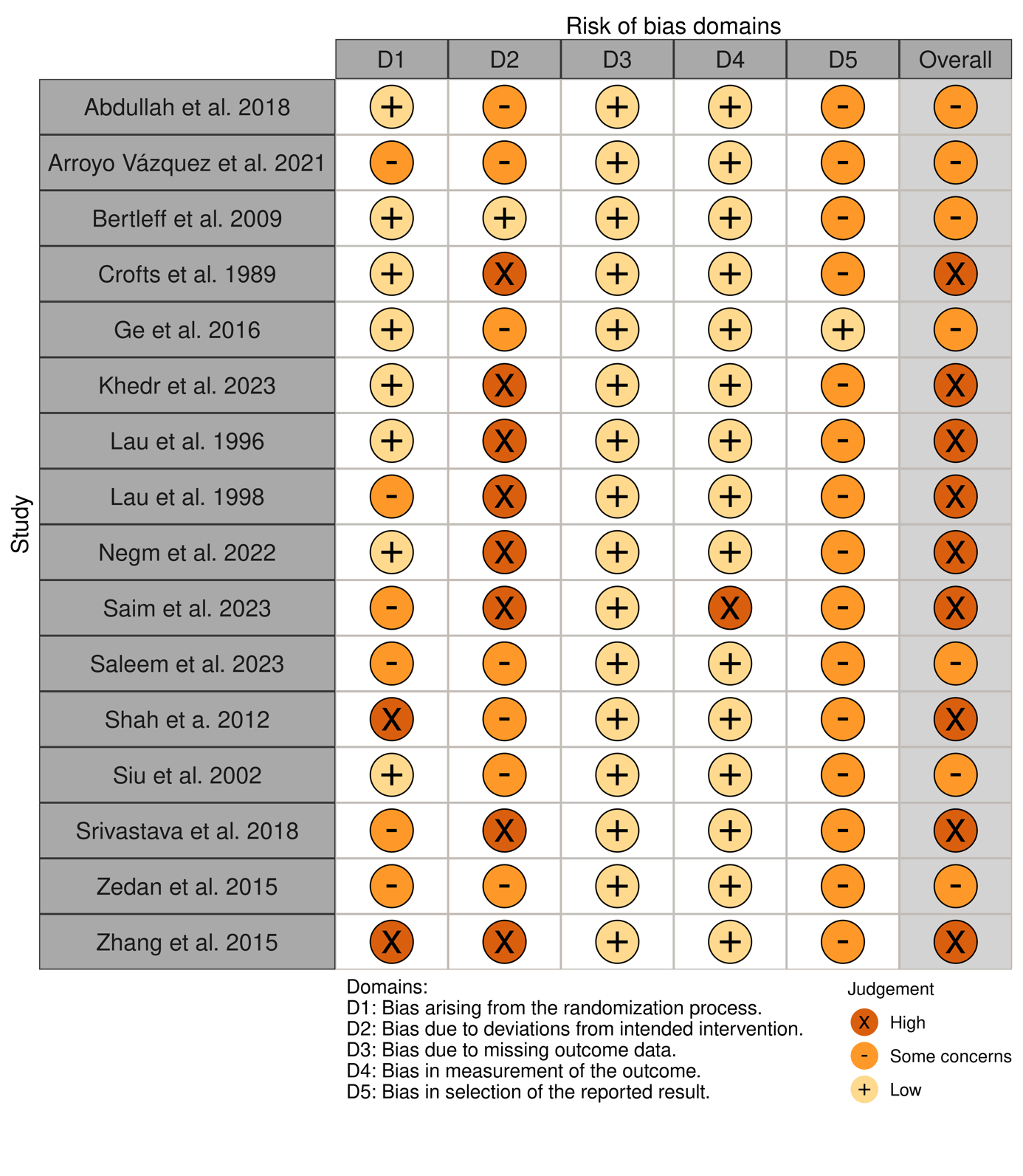


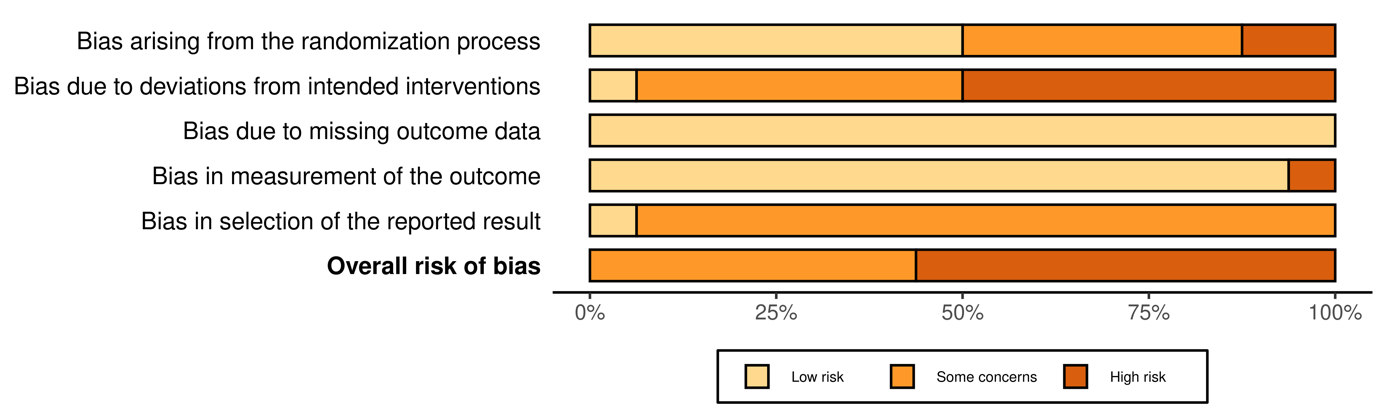


**Supplemental material 4: Risk of bias**

Supplement: Supplementary file 4 — (640KB) [file 423_2025_3848_MOESM4_ESM.docx]
